# Increased mitochondrial proline metabolism sustains proliferation and survival of colorectal cancer cells

**DOI:** 10.1371/journal.pone.0262364

**Published:** 2022-02-07

**Authors:** Saif Sattar Alaqbi, Lynsey Burke, Inna Guterman, Caleb Green, Kevin West, Raquel Palacios-Gallego, Hong Cai, Constantinos Alexandrou, Ni Ni Moe Myint, Emma Parrott, Lynne M. Howells, Jennifer A. Higgins, Donald J. L. Jones, Rajinder Singh, Robert G. Britton, Cristina Tufarelli, Anne Thomas, Alessandro Rufini

**Affiliations:** 1 Leicester Cancer Research Centre, University of Leicester, Leicester, United Kingdom; 2 Faculty of Veterinary Medicine, Department of Pathology and Poultry Diseases, University of Kufa, Kufa, Iraq; 3 Department of Cellular Pathology, University Hospitals of Leicester, Leicester, United Kingdom; 4 Leicester van Geest Multi-OMICS Facility, Leicester, United Kingdom; Hokkaido University: Hokkaido Daigaku, JAPAN

## Abstract

Research into the metabolism of the non-essential amino acid (NEAA) proline in cancer has gained traction in recent years. The last step in the proline biosynthesis pathway is catalyzed by pyrroline-5-carboxylate reductase (PYCR) enzymes. There are three PYCR enzymes: mitochondrial PYCR1 and 2 and cytosolic PYCR3 encoded by separate genes. The expression of the *PYCR1* gene is increased in numerous malignancies and correlates with poor prognosis. *PYCR1* expression sustains cancer cells’ proliferation and survival and several mechanisms have been implicated to explain its oncogenic role. It has been suggested that the biosynthesis of proline is key to sustain protein synthesis, support mitochondrial function and nucleotide biosynthesis. However, the links between proline metabolism and cancer remain ill-defined and are likely to be tissue specific. Here we use a combination of human dataset, human tissue and mouse models to show that the expression levels of the proline biosynthesis enzymes are significantly increased during colorectal tumorigenesis. Functionally, the expression of mitochondrial PYCRs is necessary for cancer cells’ survival and proliferation. However, the phenotypic consequences of PYCRs depletion could not be rescued by external supplementation with either proline or nucleotides. Overall, our data suggest that, despite the mechanisms underlying the role of proline metabolism in colorectal tumorigenesis remain elusive, targeting the proline biosynthesis pathway is a suitable approach for the development of novel anti-cancer therapies.

## Introduction

The rewiring of cellular metabolism has been recognized as a key hallmark of tumorigenesis [1]. The metabolic adaptions of rapidly proliferating cancer cells fulfil critical roles, such as managing cellular redox balance, contributing to cellular energetic needs and ensuring sufficient provision of monomeric precursors of macromolecules, such as nucleic acids and proteins [2].

Changes affecting the metabolism of non-essential amino acids (NEAAs) during carcinogenesis have been extensively documented and offer opportunities for pharmacological interventions [3]. In recent years, cancer-related adaptations of the metabolism of the NEAA L-proline have received growing attention. The metabolism of the NEAA L-proline plays a key role in cancer progression [4,5]. L-proline is synthesized from its precursor pyrroline-5-carboxylate (P5C) through the activity of NAD(P)H-dependent P5C reductases (PYCRs) (S1 Fig). There are three homologous *PYCR* genes: *PYCR1* and *2* encode for mitochondrial enzymes that share a high degree of similarity (>80%), while *PYCR3* (also known as *PYCRL*) encodes a less conserved (~45%) cytoplasmic isoform [6–8]. Moreover, mitochondrial PYCRs (mtPYCRs) enzymes are chiefly involved in processing P5C originating from the precursor L-glutamate and have higher affinity for NAD/NADH, whereas PYCR3 has been implicated in the biosynthesis of L-proline from the ornithine route and preferentially metabolize NADP/NADPH [6,7]. mtPYCRs have been widely implicated in carcinogenesis [5]. The *PYCR1* gene emerged as one of the most consistently overexpressed metabolic genes in human cancers [4,5,9,10] and its expression is associated with unfavorable prognosis in several malignancies [11–17]. Mechanistically, *PYCR1* expression sustains survival and proliferation of cancer cells [11,13,14,16,18–22]. This ability has been attributed to the capacity of the proline biosynthesis pathway to regulate key physiological functions, including protein synthesis, redox balance, ratios of pyridine nucleotides, nucleotide biosynthesis and mitochondrial function [4,5,17,23]. Moreover, PYCR1 activity has been linked to regulation of the amino acid response pathway (AAR) and the mTOR pathway [4,5]. It has also been suggested that a proline cycle enables exchange of reduced equivalents between the mitochondrial and the cytosol [24–26]. The cytosolic reduction of P5C to proline during this cycle would generate oxidized NAD(P)+ that would then stimulate production of ribose, the sugar constituent of nucleotides, through the pentose phosphate pathway (PPP) (S1 Fig) [10,26,27]. In addition to its cell-autonomous roles, proline is also a major component of collagen and therefore a key contributor to the composition of the extracellular matrix and the tumor microenvironment [4,28]. Moreover, the ability of proline biosynthesis to act as a rate-limiting pathway in collagen biosynthesis can have wider consequences, as collagen prolyl-hydroxylase have been shown to promote tumor growth in breast cancer by modulating alpha ketoglutarate and succinate levels thus reducing proline hydroxylation on hypoxia-inducible factor (HIF) 1α, enhancing its stability [29].

As the mechanistic causality linking *PYCR* genes and their expression on the one side and malignant growth on the other is not well-defined, we wished to explore it further focusing on colorectal cancer (CRC). Using a combination of analyses of human tissues and a genetically modified mouse model of CRC, we show that the expression of PYCR enzymes is upregulated during colorectal carcinogenesis. Experiments performed using genetic depletion of mtPYCR enzymes convincingly demonstrate that proline metabolism is integral to the ability of CRC cells to proliferate and survive. Moreover, our data also reveal that, in CRC, the pro-tumorigenic impact of mitochondrial PYCRs is independent of proline or nucleotide biosynthesis.

## Material and methods

### Cell culture

Cell lines were supplied by the departmental cell line bank. SW620 and CL11 were purchased from ATCC (Middlesex, UK) and DSMS (Braunschweig, Germany), respectively. Cells were cultured at 37°C in 5% CO_2_. HCEC cell line [30] were grown in Fibronectin/Collagen coated cell culture flasks. HCT116 were grown in McCoy’s 5A (Thermo Fisher, Cat#11594466). RKO and Caco2 cells were grown in MEM (Gibco, Cat#M5650). SW480, SW620, CL11, HT29 and HCEC cell lines were grown in DMEM. Media were supplemented with GlutaMAX (Gibco, Cat#36600–021) and 10% FCS (Fisher Scientific, Cat#11550356). Human plasma like medium (HPLM) [31] was purchased from Thermo Fischer (Cat# A4899101) and supplemented with 10% FCS. For proline supplemented media, the proline powder (Sigma, Cat#P5607-100g) was dissolved in culture medium with dialyzed fetal bovine serum (10%) (HyClone™, Thermo Fisher, Cat#12349822). Nucleoside supplemented media was prepared by diluting 100x EmbryoMax Nucleosides mix (Sigma, Cat#ES-008-D) (0.73 g/L Cytidine, 0.85 g/L Guanosine, 0.73 g/L Uridine, 0.8 g/L Adenosine, 0.24 g/L Thymidine) in growth medium with dialyzed fetal bovine serum (10%). For nutrient starvation cells were incubated for 1 hour in Hanks’ Balanced Salt Solution (Sigma, Cat#H9269-500ML). The Leicester Cancer Research Centre at the University of Leicester supplied the primary cells through the Leicester Experimental Cancer Medicine Centre (ECMC). Cells were isolated from patients undergoing surgical resection of CRC, as part of the ‘Development and Application of Stem Cell Assays for Prevention and Treatment of Cancer’ study, a fully anonymised, non-consented study approved by the Wales REC 4, reference #19/H0402145/, PI: Prof. W. Steward. All methods were carried out in accordance with relevant guidelines and regulations.

### Immunostaining

For immunohistochemistry (IHC), formalin-fixed, paraffin embedded (FFPE) sections of mouse tissues were stained using ultra-sensitive ABC peroxidase staining kit (Thermo Fisher, Cat#32054) according to the manufacturer’s instructions. Sections were dewaxed by incubation at 65°C for 20 min and then twice in xylene (Genta Medical, Cat#XYL050) for 3 min, followed by re-hydration in a graded series of industrial methylated spirit (IMS) (Genta Medical, Cat#I99050) (99% twice for 3 min and 95% twice for 3 min) and a wash in running tap water. Antigen retrieval was performed by microwaving in 10 mM citrate buffer (pH 6.0) for 20 min. Slides were incubated with PYCR1 antibody (Abcam, Cat#Ab103314, 1:400) overnight at 4°C. Rabbit immunoglobulin fraction (Dako, Cat#X0936) was used as negative control. Antibody binding was revealed using 3,3ʹ-diaminobenzidine (DAB) staining (Leica microsystems, Cat#RE7270-K), followed by counterstaining with Mayer’s Haematoxylin (3 min), dehydration and mounting with DPX resin. Human tissue microarrays (TMAs) (Abcam, Cat#ab178129 and ab178128) were stained via the same procedure using the Novus Biological PYCR1 antibody (Cat#NBP2-20016, 1:250). Each TMA contained 48 cores: 16 samples of normal colon tissue control and 16 CRC cases in duplicate cores.

For immunofluorescence (IF), sections were dewaxed and antigen retrieval performed as described. Slides were blocked in 5% goat serum in PBS-Triton and incubated overnight at 4°C with PYCR1 primary antibody (Abcam, Cat#Ab103314). Alexa Fluor 568-Goat anti-rabbit IgG (Thermo Fisher, Cat#A-11036) secondary antibody was applied for 1 hour at room temperature in the dark. Slides were mounted with an antifade reagent containing 4′,6-diamidino-2-phenylindole (DAPI) (Thermo Fisher, Cat#P36931) to stain nuclei.

### Image analysis

IHC images were acquired using the Hamamatsu NanoZoomer Digital Slide Scanners (Leica). For IF, slides were imaged with inverted confocal microscope IX81, FV1000 (Olympus). For mouse tissue, 50 intestinal crypts were randomly selected and the Aperion ImageScope program was used to calculate the H-score of PYCR1 level using the following formula: H-score = 1*(% of weak positive) + 2*(% of positive) + 3*(% of strong positive). For human TMAs, the PYCR1 H-score intensity was assessed by a cancer pathologist (KW). Each cancer H-score was averaged from duplicate cores. ImageJ was used for analysis of IF images.

### Small-interference (si)RNA transfection

*PYCR1* targeting and non-targeting siRNA were purchased from Dharmacon (GE Healthcare, Cat#L-012349-00-0005 and D-001810-10-05) and Ambion (Thermo Fisher, Cat#4390824 and AM4611). Custom isoform-selective siRNAs for PYCR1 (GGUUACUGUGGGUGGAAUAUU) and PYCR2 (GCAAAGUGGUGAGGAGAAAUU) were purchased from Dharmacon and designed using Horizon Discovery Custom siRNA design tool (https://horizondiscovery.com/en/ordering-and-calculation-tools/custom-sirna). Each selective siRNA targets the 3’-UTR region of the corresponding gene, which is unique to each isoform but shared between the different splice variants. Alignment of both the *PYCR1* and *PYCR2* selective siRNAs to 3’-UTR region of the respective genes was performed in SnapGene (Version 5.3.2), whereas the specificity of the custom siRNA sequences was assessed using the web-based bioinformatics tool SpliceCenter [32].

For siRNA transfection, 50,000–100,000 cells were seeded in 6 cm^2^ dishes. When reaching ~30% confluence (~48h), the cells were transfected with 100 pmol of siRNA using Lipofectamine RNAiMAX (Thermo Fisher, Cat#13778075), according to manufacturer’s instructions, for 24 to 72 hours. Briefly, on the day of transfection, siRNA was defrosted on ice and 100 pmol siRNA was added to 250 μL Opti-MEM Reduced Serum Medium (Life technologies, Ref 31985–070) in a sterile microcentrifuge tube. 10 μL of Lipofectamine RNAiMAX was added to 250 μL Opti-MEM Reduced Serum Medium in a separate microcentrifuge tube. The contents of each tube were mixed together and allowed to sit at room temperate for at least 15 minutes prior to adding the mixture to the cells in culture. The cells were then returned to the incubator for the required transfection time. For proline supplementation experiment, cells were plated, transfected and incubated in supplemented media. For nucleoside supplementation, supplemented media was added to the cells 24h post transfection and refreshed every 24 hours for the duration of the experiment.

### Detection of apoptosis

Apoptotic cells were detected using FITC-Annexin V Apoptosis Detection kit (BD Biosciences). HCT116 cells were collected 48 hours post-transfection. Floating dead and adherent live cells were pulled and washed with fresh growth medium. After centrifugation, the cell pellet was resuspended in 500 μL 1X Annexin V Binding Buffer + 5 μL FITC-Annexin V and 5 μL of Propidium Iodide and incubated at room temperature in the dark for 15 min. The percentage apoptotic cells was determined by flow cytometry (10,000 cells) using a FACS Aria II (Becton Dickinson) with the BD FACS Diva analysis software (version 6.1.2). Positive control HCT116 cells were treated with 25 μM Etoposide for 48 hours to induce apoptosis.

### Measurement of proliferation

72 hours post-transfection, cells were pulsed with 10 μM EdU for 1 hour and EdU staining was performed using the Click-iT® EdU Alexa Fluor® 488 Flow Cytometry Assay (Thermo Fisher, Cat#C10337), according to manufacturer’s instructions. Total DNA staining was performed using Fx Cycle™ Violet stain (Thermo Fisher, Cat#F10347). Analysis of total DNA (excitation 405 nm, emission 450/50 band-pass) and EdU (excitation 488 nm, emission 530/30 nm) was undertaken by flow cytometry using BD FACS ARIA II (Becton Dickinson) with the BD FACS Diva analysis software.

### Western blotting

**Fig 1:**
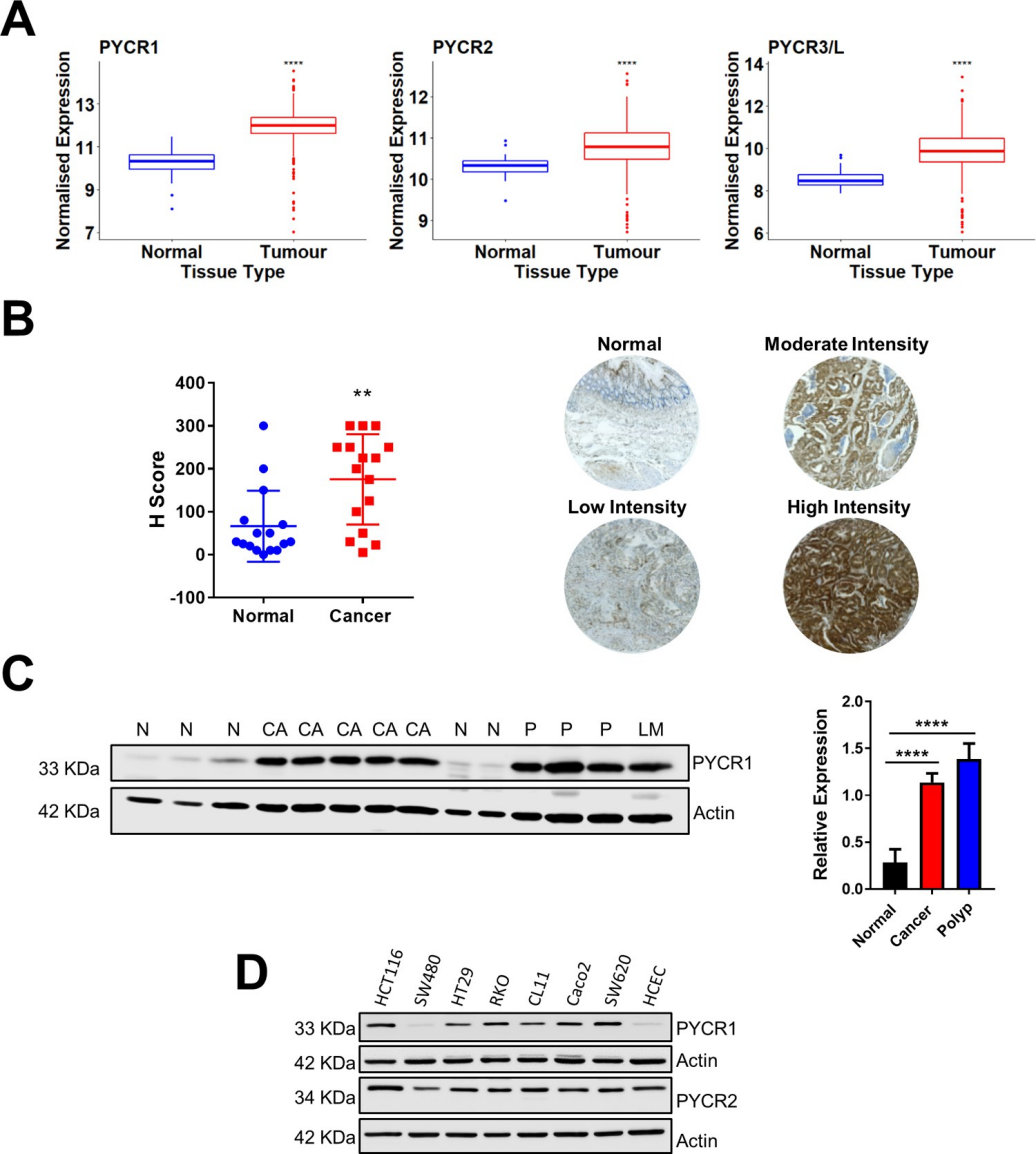
Expression of *PYCR* genes in human CRC. **A)** Bioinformatics analysis showing increased expression of *PYCR1*, *PYCR2* and *PYCR3* in the TCGA CRC dataset (n = 41 normal and 41 cancers). Data were analyzed using unpaired *t*-test. **** p<0.0001. **B)** PYCR1 H-score was assessed in one CRC TMA. Each dot represents a single core for normal specimens and the average of two separate cores for CRC cases. Lines indicate mean ± SD and data were analyzed by two-tailed *t*-test (n = 16 normal and 16 cancers). ** p<0.01. Representative histo-spots are shown. **C)** PYCR1 expression was analyzed by western blotting in lysate from cell suspensions isolated from primary colon polyps (P), carcinomas (CA), liver metastasis (LM) and, when available, matched normal tissues control (N). Actin was used as endogenous loading control. The relative expressions was calculated relative to the normal group using ImageJ software and plotted as mean ± SD. Data were analyzed using One-way ANOVA and Dunnett’s multiple comparisons test (n = 5 normal tissue controls, 6 CRC cases [including 1 liver metastasis] and 3 benign polyps). **** p<0.0001. N = normal, CA = cancer and LM = liver metastasis. **D)** Expression of proline metabolism enzymes in the indicated CRC cell lines by western blotting. Actin was used as endogenous loading control.

Samples were processed as described previously [33]. Nitrocellulose membranes were incubated overnight at 4°C with primary antibodies. The following day, the membrane was washed three times in PBS-Tween and incubated for 1 hour at room temperature with secondary antibodies (1:10,000). Enhanced Chemiluminescence Luminol (ECL) solution (Geneflow Ltd, Cat#K1-0170) was used to detect protein either through exposure to X-ray film (Thermo Scientific, Cat#4351379) developed using an Agfa Curix 60 developer, or the GeneGnome XRQ machine (Syngene) with GeneGnome XRQ software. All antibodies are listed in S1 Table. Quantitation of western blot bands in Fig 1C was performed using ImageJ.

### Real-time (rt)PCR

RNA was isolated using the RNeasy mini kit (Qiagen, Cat#74104), following manufacturer’s instructions. RNA concentration was measured with the NanoDrop spectrophotometer by averaging two readings at 260 nm. 2 μg of RNA were retrotranscribed using RNA-to-cDNA kit (Applied Biosystems, Thermo Fisher, Cat#4388950). TaqMan® probe technology was used to analyze gene expression by rtPCR (S2 Table). 10 μL of TaqMan® Gene Expression Master Mix (Applied Biosystems) was mixed with 1 μL of target-specific TaqMan FAM-MGB probe, 1 μL of housekeeping endogenous control TaqMan VIC-MGB probe, 2 μL of cDNA and 6 μL of water. PCR were run using Applied Biosystems Step One Plus machine (holding stage 95°C for 20 seconds, 40 cycles at 95°C for 1 seconds and 60°C for 20 seconds). The comparative cycle threshold C_T_ method was used for analysis. All samples were run in triplicate and the mean values of C_T_ were used for calculating the ΔC_T_. Normalization was performed using geometric mean of two endogenous genes, *Pop4* and *Efnb2*, identified through the NormFinder method [34].

### Detection of L-proline

#### Liquid chromatography-mass spectrometry (LC-MS)

Cells were washed with 0.9% sodium chloride solution (NaCl: Sigma, Cat#S7653-250G, Water: Fisher, Cat#W/0112/17) and frozen immediately on dry ice. Metabolites were extracted in ice cold solvent (1:1.5:1.5 H_2_O:acetonitrile:methanol) and analyzed by HILIC LC-MS at the Birmingham Phenome Centre metabolomics facility at the University of Birmingham. Data were processed by XCMS. PQN was used to normalize data prior to statistical analysis. Peaks were annotated using the Human Metabolome Database (HMDB) and comparison to a library of standards using m/z and retention times. Peaks were further confirmed using online databases (mzCloud) when MS/MS spectra were available.

#### Acid-Ninhydrin detection of L-proline

2 million cells were reconstituted in 300 μL extraction buffer (Elabscience, Caltag medsystems, Cat#E-BC-K177 Reagent 1) and freeze-thawed twice before analysis. Fresh 2.5% ninhydrin solution was prepared by dissolving 0.25 g ninhydrin (Sigma, ACS reagent, Cat#151173 -25G) in 10 mL Glacial Acetic Acid (Fisher Chemical, HPLC grade, Cat#A/0406/PB15). Proline standards were prepared using L-proline (Sigma, Cat#P0380) with extraction buffer as solvent. 300 μL sample/standard was added to 600 μL of the ninhydrin solution and boiled for 30 min. 200 μL of each solution was transferred into a 96-well plate in triplicate and measured at 508 nm.

### NADPH/NADP fluorometric assay

The NADPH/NADP assay (Abcam, Cat#ab65349) was performed as per the manufacturer’s protocol. Pellets of 5x10E6 cells were washed with cold PBS and dissolved in 800 μL extraction buffer. Samples were subjected to two freeze/thaw cycles (20 minutes on dry ice followed by 10 minutes at room temperature). Samples were then vortexed for 10 seconds and centrifuged at high speed for 5 minutes and kept on ice. 10 kD spin columns (Abcam, Cat#ab93349) were then used to remove enzymes that consume NADPH. DNA was thoroughly sheered with needle and syringe. For total NADP and NADPH, samples were left as prepared. For selective quantification of NADPH, 200 μL of sample was transferred to a separate microcentrifuge tube and heated to 60˚C for 30 minutes. Samples were then cooled on ice until analysis. A set of standards (0, 20, 40, 60, 80, 100 pmol/well) were freshly prepared from a 1 mM NADPH stock solution. 50 μL of either sample or standard were added to a 96 well plate. 100 μL of the Reaction Mix (containing 98 μl NADP Cycling Buffer and 2 μl NADP Cycling Enzyme mix) was added to each well other than the wells for background calculation, to which 100 μL of Background Reaction mix (containing only NADP Cycling Buffer) was added. The plate was left for five minutes at room temperature before 10 μL of NADPH Developer solution was added into each well and mixed by pipetting up and down. The plate was then left at room temperature for 1 hour. Absorbance was read at 450 nm with a Fluostar Optima plate reader (BMG Labtech LTD).

### *In vivo* study

Animal work was performed under the project license (PPL) no. 60/4370, granted by the Home Office/UK and reviewed by the local Animal Welfare Ethical Review Body (AWERB). Animals, hosted in the specific-pathogen-free Pre-clinical Research Facility (University of Leicester), were fed *ad libitum* with AIN93 diet (TestDiet, USA, Cat#5801-G), under a climate-controlled environment with 12 hours day/night cycle. *LGR5-Cre*^*ER+*^*/Apc*^*fl/fl*^ (with expression of Cre recombinase) and control *LGR5-Cre*^*ER-*^*/Apc*^*fl/fl*^ (Cre negative) C57BL6/J mice of both sexes aged 2–3 months were used in this study. To induce loxP recombination, mice were intraperitoneally injected with 3 mg of tamoxifen (Sigma, Cat#T5648) (10 mg/mL tamoxifen in sunflower oil). Animal were randomly allocated to three groups and sacrificed under terminal anesthesia with cervical dislocation at three days, one week and two weeks post tamoxifen injection. The small intestine was harvested, washed with PBS, and snap-frozen in liquid nitrogen for RNA purification or swiss-rolled and fixed overnight in 4% paraformaldehyde for IHC processing. Study groups were not based on power calculations and experimenters were not blinded to the randomly allocated treatment groups.

### Statistical analysis

Statistical analysis was carried out using GraphPad Prism or R Studio. Student’s *t*-test and ANOVA, with *post hoc* correction for multiple hypothesis testing, were applied for group comparisons. A p-value ≤ 0.05 was considered statistically significant. Analysis of the COAD (colon adenocarcinoma) TCGA dataset [35] was completed using RStudio (R version 4.0.2). Paired normal and tumor tissue sample HTSeq data was extracted using the TCGAbiolinks_2.16.4 package and normalized using the Variance Stabilizing Transformation function from DESeq2_1.28.1. Graphs were plotted using ggplot2_3.3.3 boxplots and stat_compare_means function used to apply *t*-test analysis. Survival analysis was completed using ‘limma-pipeline’ package (limma_3.44.3).

## Results

### Expression of *PYCR* genes is increased in CRC

To investigate whether the gene expression levels of proline metabolism enzymes are altered in CRC, we interrogated the TCGA transcriptomic dataset (Figs 1A and S2A) [35]. When compared to normal tissue, CRC displayed significant increased expression of *PYCR1* gene, as well as *PYCR2* and *PYCR3* genes (Fig 1A) and no significant changes in PRODH levels (S2A Fig). Analysis of two commercially available TMAs containing both normal and CRC tissues confirmed accumulation of PYCR1 enzyme in cancer tissue (Figs 1B, S2B and S2C). Indeed, over 50% of CRC cores scored high for PYCR1 expression versus only 12% high scorers in normal tissue controls (S2C Fig). Accumulation of the PYCR1 protein was also confirmed by western blot analysis performed on primary cells isolated from surgically resected colon neoplasia and adjacent normal tissue, as well as by assessment of a panel of CRC cancer cell lines (Fig 1C and 1D). When compared to control human colon epithelial cells (HCEC), most CRC cell lines showed increased expression of PYCR1.

To assess whether the expression of proline metabolism genes has prognostic relevance in CRC, we investigated survival of patients with colon adenocarcinoma from the TCGA dataset. Whereas no association was identified between patients’ survival and expression levels of *PYCR1* and *PYCR3*, increased expression of *PYCR2* was associated with worse prognosis in patients with stage 4 disease (S3 Fig).

**Fig 2:**
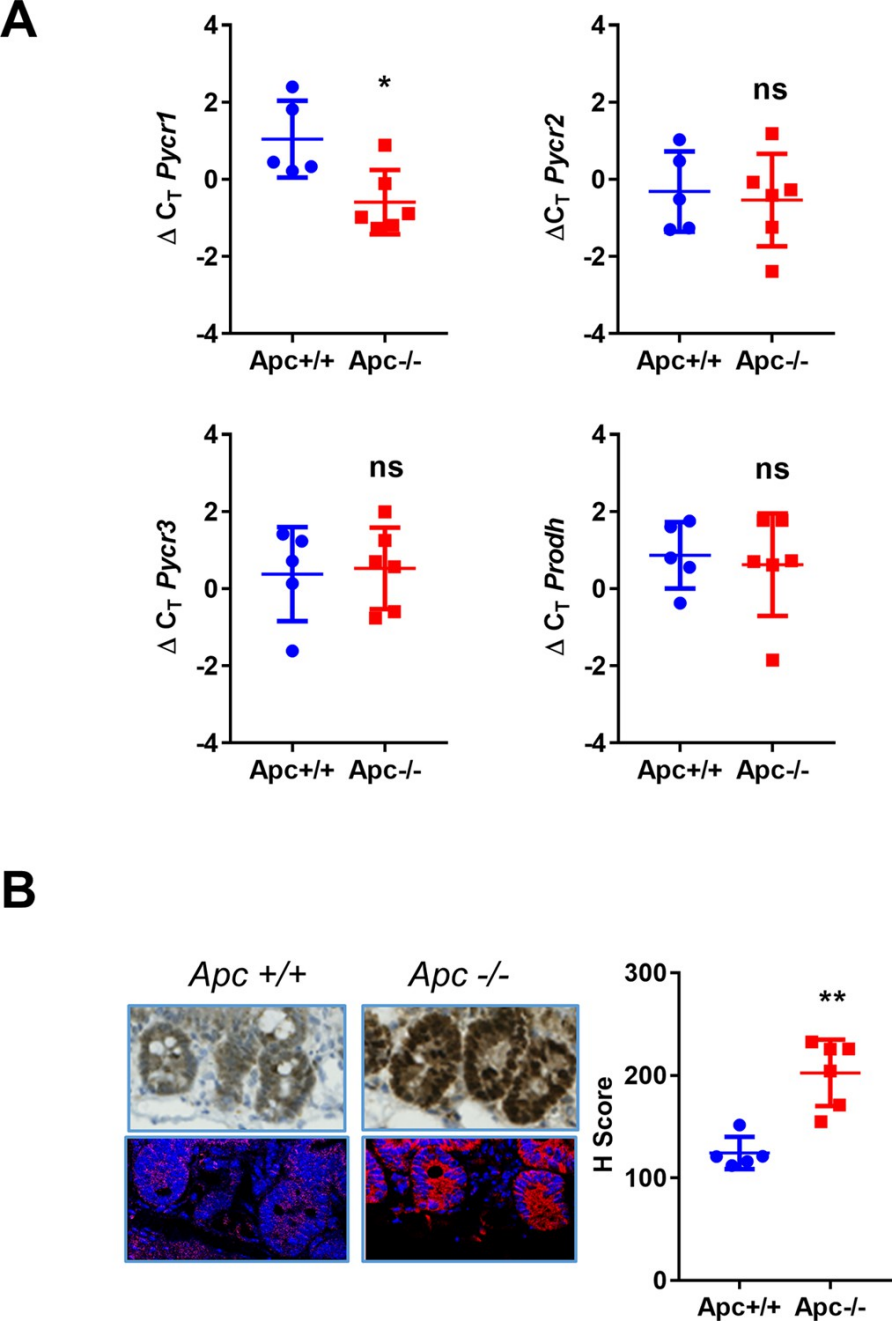
Expression of proline metabolism enzymes in a mouse model of CRC. **A)** mRNA levels of the indicated genes were measured in *Lgr5-Cre*^*ER*^*/Apc*^*fl/fl*^ mice two weeks after tamoxifen injection. The scatter blots represent the ΔCT values in *Apc* deleted (*Apc-/-*) mice and *Apc* WT (*Apc+/+)* controls. Hence, a decrease in ΔCT is equivalent to an increase in gene expression. Each dot represents one mouse, and horizontal bars indicate mean ± SD. Normalization was obtained with the geometric mean value obtained from two endogenous housekeeping genes, *Pop4* and *Efnb2*. Data were analyzed using two-tailed *t*-test (n = 5 *Apc* WT controls and 6 *Apc* deleted mice). * p≤0.05, ns = p>0.05. **B)** Representative IHC and fluorescence staining and H-score quantification of Pycr1 protein in the small intestinal crypts of the *Lgr5-Cre*^*ER*^*/Apc*^*fl/fl*^ mice two weeks after tamoxifen injection. Each dot represents one mouse (50 crypts were scored for each animal and the average H-score is plotted), horizontal bars indicate mean ± SD. Data were analyzed using two-tailed *t*-test (n = 5 *Apc* WT controls and 6 *Apc* deleted mice). ** p<0.01.

Next, we used the *Lgr5-Cre*^*ER*^*/Apc*^*fl/fl*^ mouse model of CRC [36], where expression of 4-hydroxytamoxifen-dependent Cre recombinase is regulated by the endogenous *Lgr5* gene promoter and is restricted to the intestinal stem cells located at the bottom of the crypt [37]. Tumorigenesis is triggered by Cre-mediated depletion of a floxed *Apc* allele following intraperitoneal injection of tamoxifen, leading to constitutive activation of the Wnt signaling pathway. *Lgr5-Cre*^*ER*^*/Apc*^*fl/fl*^ mice were treated with tamoxifen and culled three days, one week and two weeks after to assess the expression of *Pycr* genes in intestinal tissue. rtPCR analysis revealed no increase in *Pycr1* mRNA expression in intestinal tissue harvested from mice three days or one week after depletion of the tumor suppressor *Apc*, but accumulation of the *Pycr1* transcript and protein in intestinal tissue became evident two weeks post *Apc* depletion (Figs 2A, 2B and S4). No alterations in the expression levels of *Pycr2*, *Pycr3* and *Prodh* were recorded by rtPCR (S4 Fig).

Overall, these data indicate that proline biosynthesis enzymes are upregulated in CRC and have potential prognostic significance.

### Expression of *mtPYCR* genes is necessary for the proliferation of CRC cells

**Fig 3:**
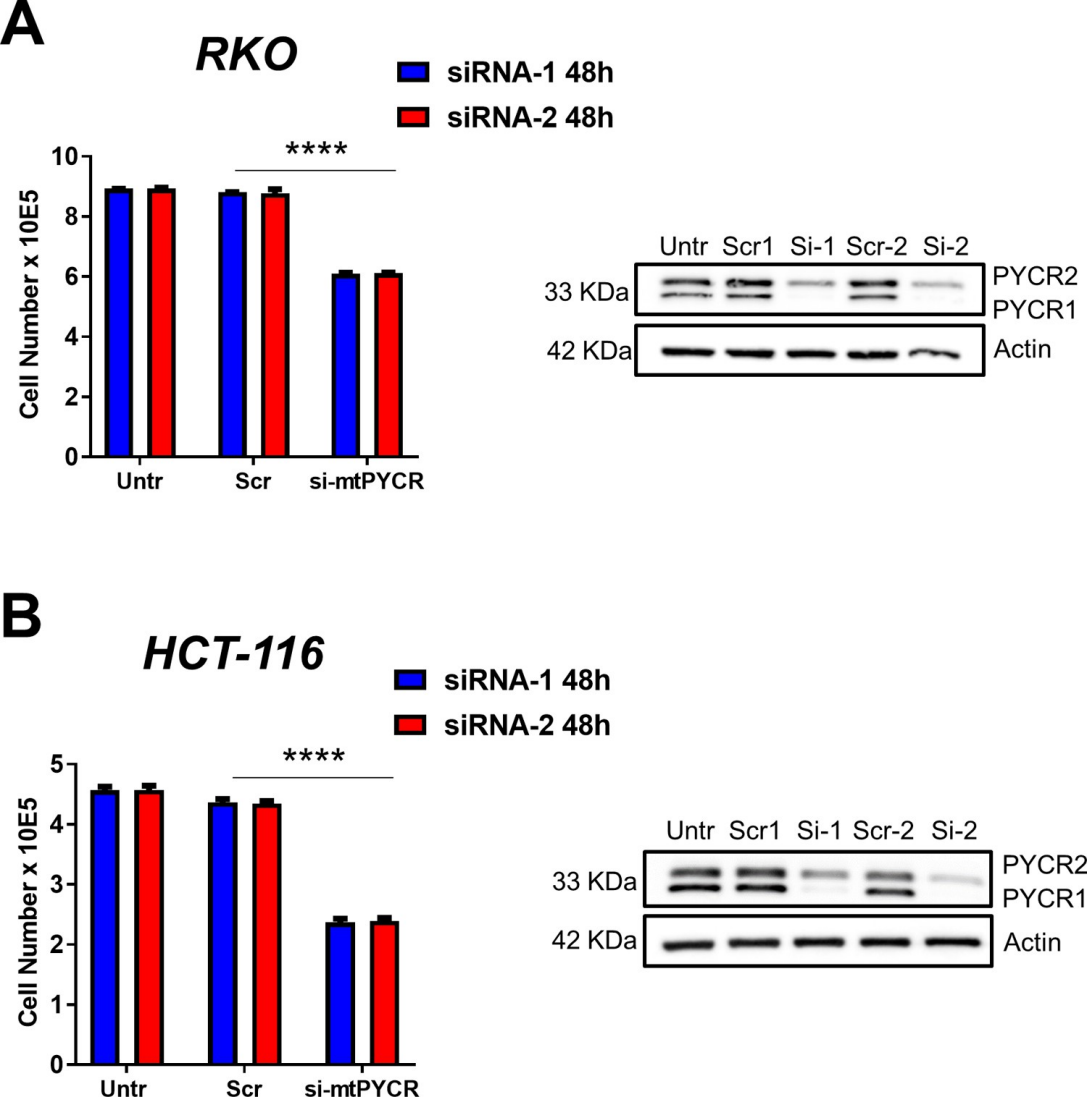
Knock-down of *PYCR1* decreases growth of CRC cell lines. Bar graphs showing number of RKO **A)** and HCT116 **B)** cells 48 hours after transfection with two independent siRNAs (siRNA-1 Dharmacon, siRNA-2 Ambion) targeting *PYCR1* (si-mtPYCRs) and control siRNA (Scr). Untr indicates control cells without any treatment. The representative western blot shows the PYCR1 and PYCR2 protein expression in the corresponding samples. Actin was used as loading control. In this experiment the same nitrocellulose membrane was sequentially incubated with the PYCR1 (Thermo Fischer) antibody and the PYCR2 (Atlas) (S1 Table). The bars represent mean ± SD. Data were analyzed using Two-way ANOVA and Tukey’s multiple comparisons test (n = 3 independent experiments). **** p<0.0001.

To investigate the functional relevance of *PYCR1* in CRC, we used two commercially available small interfering RNAs (siRNA) to deplete *PYCR1* expression in CRC cell lines. A 48h treatment with either of the two siRNAs against *PYCR1* resulted in a significant reduction in cell number in RKO (Fig 3A) and HCT116 (Fig 3B) CRC cell lines. This reduction in cell number correlated with a robust decrease in PYCR1 protein expression (Fig 3A and 3B). Unexpectedly, we also observed a decrease in the homologous PYCR2 protein in cells treated with *PYCR1*-targeting siRNA. To acknowledge the lack of specificity of the tested siRNAs, henceforth we will refer to treated cells as si-mtPYCR (or siPYCR). Moreover, since both siRNAs behaved similarly, we used siRNA-1 for the following experiments.

We selected SW620 as a third experimental cell line to validate the impact of genetic depletion of mtPYCRs on cellular growth. We confirmed that siRNA-treated cells displayed a compromised cell growth associated with reduced expression of both *PYCR1* and *PYCR2*, but no changes in *PYCR3* transcript levels (S5A and S5B Fig).

Traditional culture media force non-physiological metabolic adaptations in cultured cells that can bias the outcome of experimental observations [31,38,39]. To exclude the possibility that the reduced cell growth following knockdown of mtPYCRs resulted from an artifact of the *in vitro* culture media, we knocked down mtPYCRs in RKO cell grown in human plasma like medium (HPLM) [31] and again we observed a significant reduction in cell number 72h post-transfection (S5C Fig).

Finally, to ascertain whether addiction to mtPYCR expression is a distinctive feature of cancerous cells, we tested the impact of mtPYCR depletion on HCEC control cell line. Although PYCR1 protein expression in HCEC cells is marginal (Fig 1D), siRNA transfection achieved an additional reduction in PYCR1 protein levels without affecting HCEC cell growth (S5D Fig).

In summary, these data show that the expression of the mitochondrial isoforms of PYCR enzymes is necessary for the growth of CRC cells.

### Depletion of *mtPYCR* genes triggers cell cycle arrest and apoptosis

**Fig 4:**
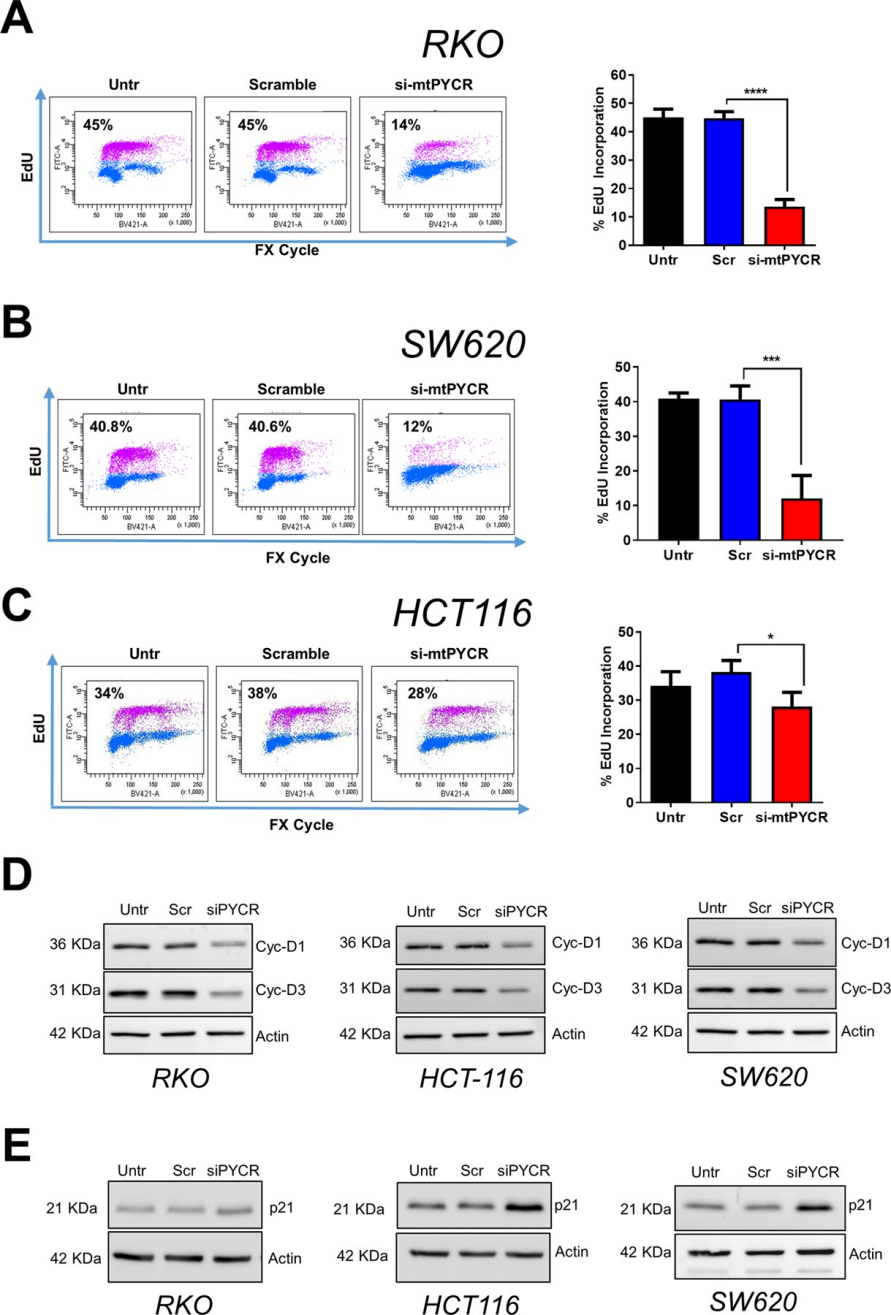
*PYCR1* depletion leads to reduced cellular proliferation. Representative flow cytometry scatterplots of EdU incorporation in RKO **A)**, SW620 **B)** and HCT116 **C)** CRC cell lines 72 hours after transfection with the indicated siRNAs. Untr indicates control cells without any treatment. EdU was measured using the Click-iT® EdU Alexa Fluor® kit and total DNA stained using FxCycle™ Violet Stain. The bar graph shows mean ± SD quantification of EdU incorporation. Data were analyzed using One-way ANOVA and Tukey’s multiple comparisons test (n = 3 independent experiments). * p≤0.05, *** p<0.001, **** p<0.0001. **D)** Western blot analysis of cell cycle markers cyclin D1 and Cyclin D3 in the indicated cell lines treated as in A-C. Actin was used as loading control. **E)** Western blot analysis of cell cycle marker p21 in the indicated cell lines treated as in A-C. Actin was used as loading control.

Next, we sought to investigate the mechanism responsible for the reduced cell growth observed in siRNA-treated CRC cells. Towards this end, we assessed changes in cellular proliferation and expression of cell cycle biomarkers following mtPYCRs knockdown. In agreement with the reported reduction in cell number, the EdU incorporation assay revealed a significant decrease in cellular proliferation 72 hours after siRNA transfection in all three CRC cell lines. In particular, RKO and SW620 cells suffered a marked reduction in EdU incorporation of about 70% (Fig 4A and 4B), whereas HCT116 exhibited a lesser decrease (Fig 4C). Moreover, the reduced proliferation was accompanied by coherent changes in the expression of cell cycle biomarkers, as indicated by the reduced expression of cyclin D1 and cyclin D3 and the increased expression of the cell cycle inhibitor protein p21 in mtPYCR-depleted cells (Fig 4D and 4E).

**Fig 5:**
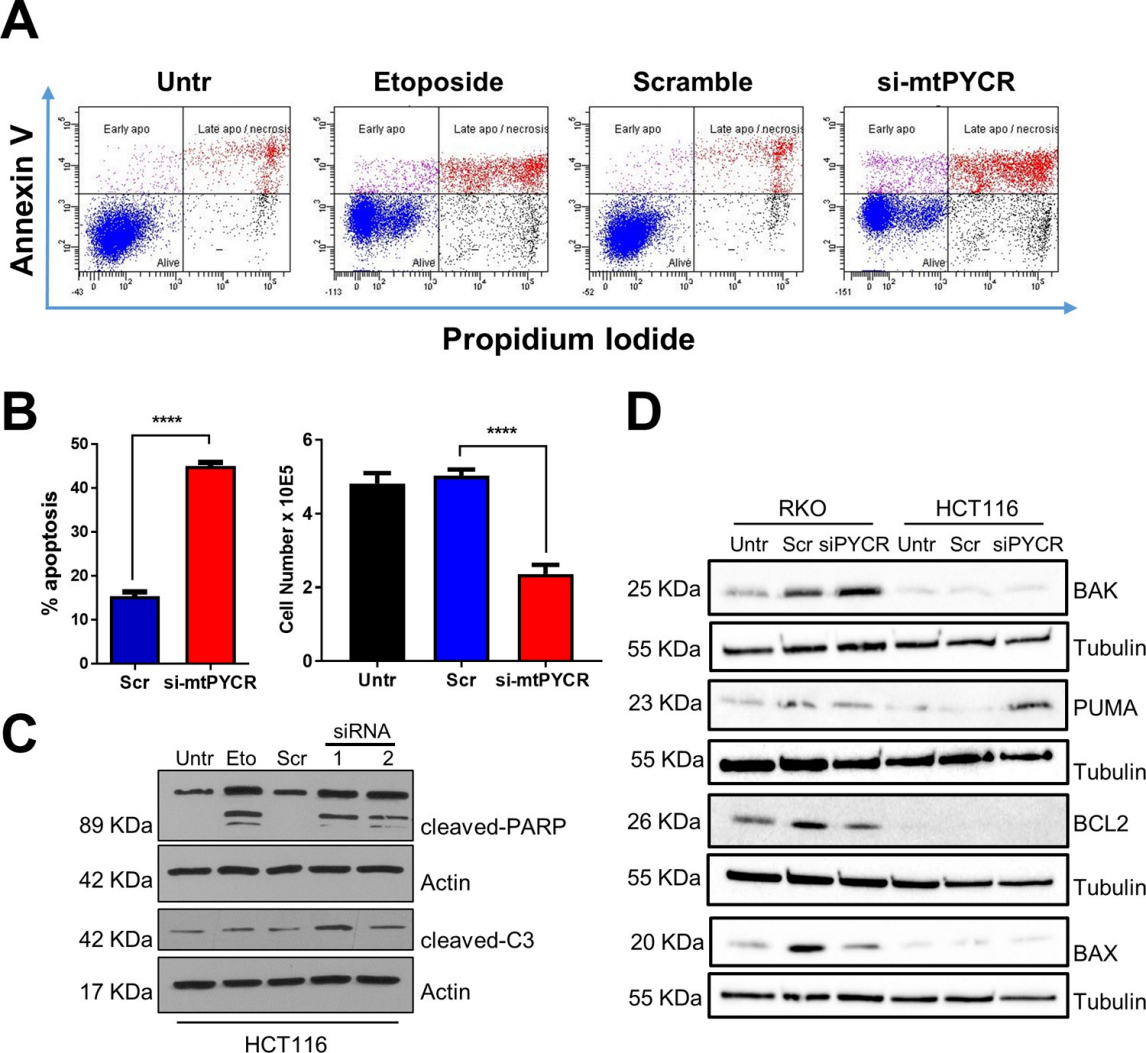
Knock-down of *PYCR* genes induces apoptosis. **A)** Representative scatter plots of cytofluorimetry-based assessment of Annexin V/PI staining in HCT116 cells transfected with siRNA targeting *PYCR* genes (si-mtPYCR) and with non-targeting scrambled siRNA (Src) for 48 hours. Untr indicates control cells without any treatment. Etoposide (25 μM) was used as positive control for induction of apoptosis. **B)** The bar graph quantification of apoptotic cell death and total cell number collected at the end of the experiment. Data are plotted as mean ± SD of three independent experiments, each performed in triplicate. **** p<0.0001, unpaired t-test or one-way ANOVA with Tukey’s multiple comparisons. **C)** Western blots showing increased cleaved-Caspase 3 and cleaved-PARP expressions following 48 hours of *PYCR1* knockdown with two independent siRNAs compared to scramble (Scr) transfected and untransfected controls (Untr). Cells treated with etoposide (25 μM) were used a positive control for induction of apoptosis. Actin was used as loading control. **D)** Western blots showing increased PUMA expression in HCT116 cells following a 72 hour *PYCR*1 knockdown. Tubulin was used as loading control. A representative of blot of two biological replicates is shown.

Visual inspection of cell cultures revealed accumulation of floating cells in HCT116 cells treated with siRNA targeting mtPYCRs, suggesting that these cells underwent cell death. Annexin V-PI staining confirmed that depletion of mtPYCRs led to a sharp increase in apoptotic cells (Fig 5A and 5B). Induction of apoptosis was further substantiated by accumulation of the selective apoptotic biomarkers cleaved-PARP, cleaved-Caspase 3 (Fig 5C) and PUMA (Fig 5D).

Overall, our data convincingly demonstrate that expression of mitochondrial PYCR sustains proliferation and survival of CRC cells.

### The pro-growth activity of mtPYCRs is not restricted by proline or nucleotide

**Fig 6:**
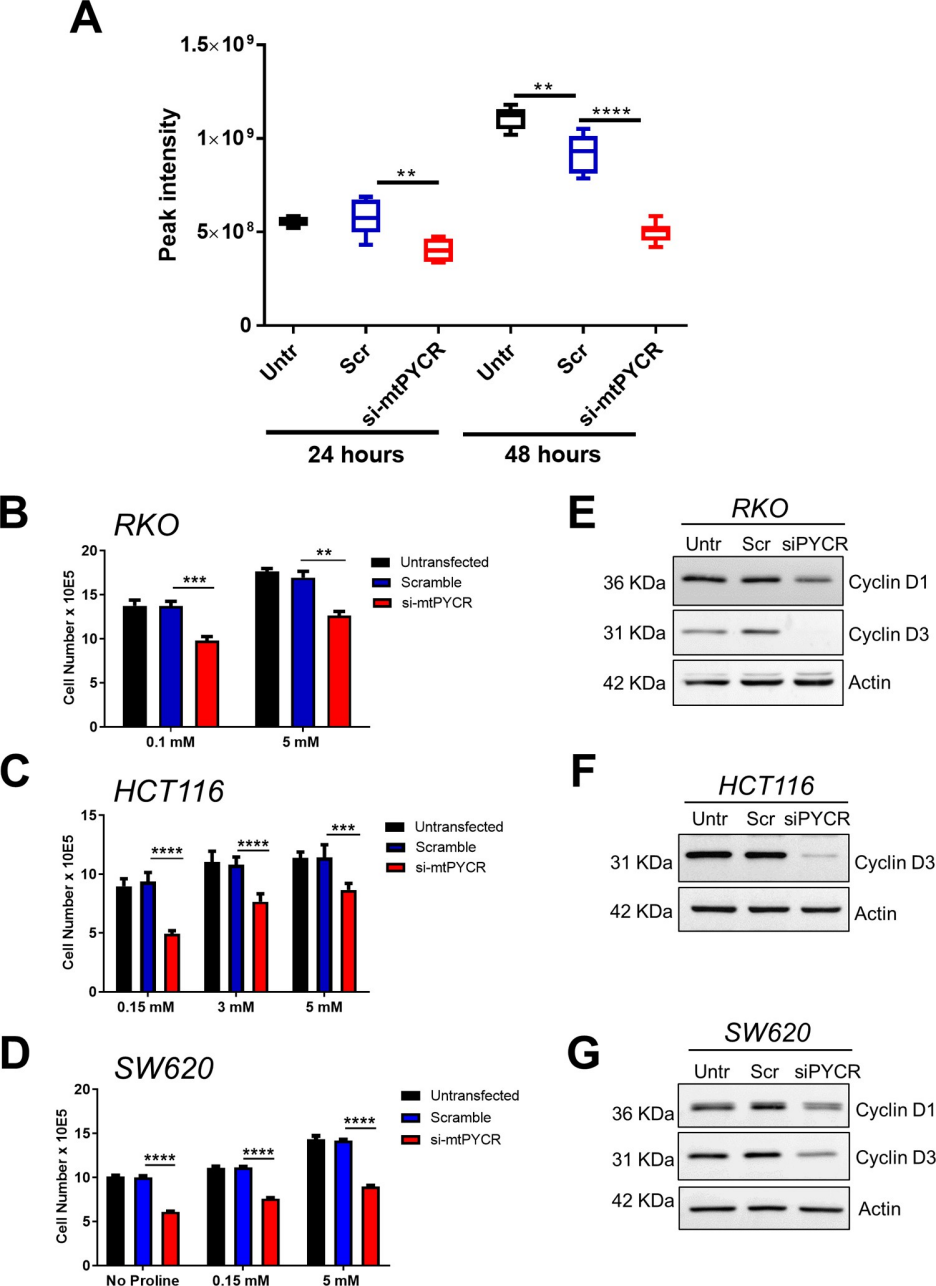
Knock-down of *PYCR* genes effect is independent of proline and nucleotide biosynthesis. **A)** Intracellular proline levels measured by HILIC mass spectrometry show decreased proline levels in RKO cells 24 hours and 48 hours after *PYCRs* knockdown. Boxes represents median and interquartile. Bars indicates highest and lowest values and were analyzed using Two-way Anova with Dunnet multiple comparisons test versus scramble control (n = 6 biological replicates). ** p<0.01, **** p<0.0001. **B)** Bar graph showing the effect of different proline concentrations on the growth of RKO cells transfected with siRNA targeting mtPYCRs (Red), non-targeting scramble control (Blue) or left untransfected cells (Black). Cells were collected 72 hours after transfection for counting. **C)** Bar graph showing the effect of different proline concentrations on the growth of HCT116 cells transfected with siRNA targeting mtPYCRs (Red), non-targeting scramble control (Blue) or left untransfected cells (Black). Cells were collected 72 hours after transfection for counting. The different proline concentrations in non-supplemented cultures reflect the starting proline content of the culture media: MEM for RKO, McCoy’s 5A for HCT116 and DMEM for SW620. **D)** Bar graph showing the effect of different proline concentrations on the growth of SW620 cells transfected with siRNA targeting mtPYCRs (Red), non-targeting scramble control (Blue) or left untransfected cells (Black). Cells were collected 72 hours after transfection for counting. Cell numbers in are plotted as mean ± SD and were analyzed using two-way ANOVA with Sidak’s multiple comparisons test (n = 3 independent experiments. ** p<0.01, *** p<0.001, **** p<0.0001. **E)**, **F)**, **G)** Western blots showing decreased expression of cyclin D1 and D3 protein expression after 72 hours mtPYCRs silencing in RKO (E), HCT116 (F) and SW620 (G) cells supplemented with 5 mM proline.

LC-MS measurements indicate that RKO cells double their intracellular content of proline over 24 hours and this increment is blunted in cells depleted of mtPYCR enzymes (Figs 6A and S6A). Hence, we reasoned that L-proline depletion could underlie the decreased growth and viability of CRC cells. To test this possibility, we supplemented the culture medium with excess proline. Proline supplementation improved cell growth in all tested conditions, but it failed to revert the reduction in cell number following mtPYCRs loss, as indicated by a proportionally similar reduction in cell number (Fig 6B–6D) and a reduction in cell cycle biomarkers similar to that observed in standard proline medium (Fig 6E–6G). This inability of externally supplemented proline to rescue cell growth was not caused by inadequate amino acid uptake, as we recorded a marked increase in intracellular proline concentration in the supplemented cultures (S6B Fig). In addition, we did not observe any change associated with amino acid-related pathways mTOR and AAR (S6C Fig).

Because of the association between proline metabolism and nucleotide biosynthesis [23,26], we decided to test whether mtPYCR depletion resulted in insufficient nucleotide supply to cancer cells. However, external supplementation of nucleosides failed to rescue cell growth. Furthermore, the NADP/NADPH ratio, which is critical in dictating the rate of the PPP [40], was unaffected by knockdown of mtPYCR in RKO cells (S6E Fig).

### *PYCR1* and *PYCR2* are both necessary for growth of CRC cells

**Fig 7:**
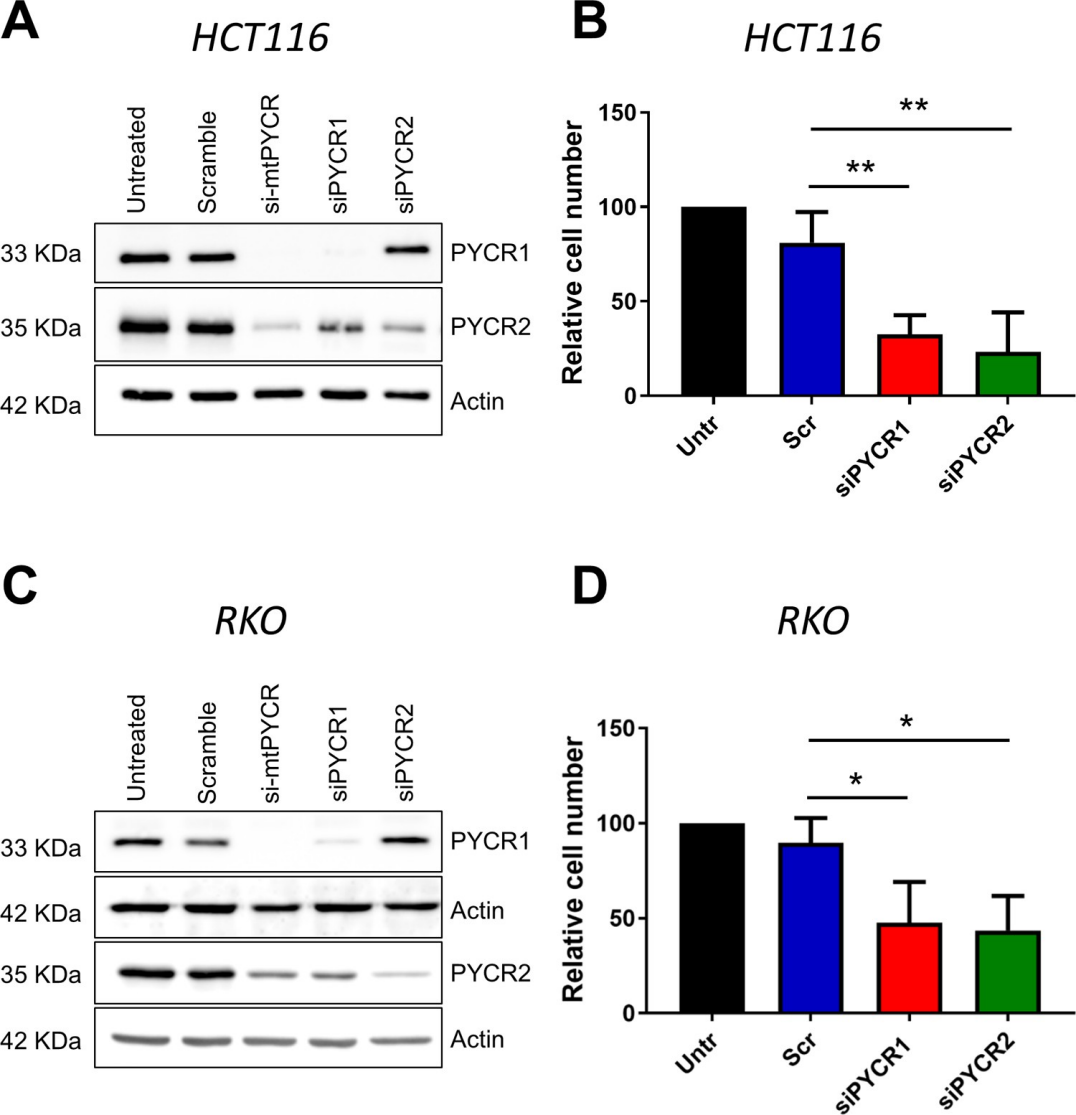
Selective isoform knockdown reveals the essentiality of both *PYCR1* and *PYCR2* expression. **A)** Representative western blot showing expression of PYCR1 and PYCR2 in HCT116 cells left untreated or transfected for 72 hours with the indicated siRNAs. Actin was used as loading control. The same membrane was blotted with all 3 antibodies PYCR1, PYCR2 and Actin. **B)** Bar graphs showing number of HCT116 cells 72 hours after transfection with custom siRNAs targeting *PYCR1* or *PYCR2* and control siRNA (Scr). Untr indicates untreated control cells. **C)** Representative western blot showing expression of PYCR1 and PYCR2 in RKO cells left untreated or transfected for 72 hours with the indicated siRNAs. Actin was used as loading control. PYCR1 and 2 were assessed in separate nitrocellulose membrane with their respective loading control. **D)** Bar graphs showing number of RKO cells 72 hours after transfection with custom siRNAs targeting *PYCR1* or *PYCR2* and control siRNA (Scr). Untr indicates untreated control cells. The bars represent mean ± SD. Data were analyzed using One-way ANOVA with Dunnet multiple comparisons test versus scramble control (n = 3 independent experiments). * p≤0.05, ** p<0.01.

Finally, we attempted to investigate the contribution of each mitochondrial isoform to colorectal carcinogenesis through the design of selective siRNAs sequences targeting the least conserved 3’UTR regions of the *PYCR1* and *PYCR2* transcripts. Western blot analysis of PYCR protein expression confirmed that the siRNA targeting *PYCR2* was selective, leaving expression of the PYCR1 protein unaffected. On the other hand, knockdown of *PYCR1* also resulted in partial loss of PYCR2 expression, indicating an epistatic dependence between the two isoforms (Fig 7A and 7C). However, we cannot formally rule out that the decreased expression of PYCR2 is the result of an off target effect of the isoform selective siRNAs. Nonetheless, depletion of either *PYCR1* or *PYCR2* resulted in a similar decrease in cell number in RKO and HCT116 CRC cell lines (Fig 7B and 7D), suggesting that the two isoforms are both necessary for the full growth of CRC cells.

## Discussion

Therapeutic options for CRC are limited by pervasive tumor heterogeneity [35,41,42]. The identification of metabolic vulnerabilities could facilitate the development of novel therapeutic approaches with broad spectrum efficacy. Interventions targeting NEAA in CRC have proved successful in pre-clinical models [33,43]. Here, we confirmed and expanded previous evidence that expression of proline biosynthesis enzymes is increased in CRC [18]. Moreover, in agreement with recent findings [44], we identified an association between expression of *PYCR2* gene and decreased survival in patients with stage IV disease. When using a mouse model of CRC, we also showed that the rewiring of proline metabolism occurs early during colorectal tumorigenesis. Indeed, we detected significantly increased *Pycr1* expression two-week post depletion of the tumor suppressor gene *Apc* in the mouse intestine, when no intestinal adenoma has yet developed. This observation is in agreement with data indicating an early, *Myc*-dependent metabolic rewiring of human CRC [45]. Moreover, tumor development in the *Lgr5-Cre*^*ER*^*/Apc*^*fl/fl*^ model depends on the activity of the transcription factor Myc [46] and *PYCR1* is a Myc target gene [47], suggesting that the enhanced expression of *PYCR1* in CRC plausibly results from Myc transcriptional activity. Nonetheless, the analysis of the TCGA dataset suggested that all *PYCR* genes are upregulated in CRC tissue, whereas our mouse study only showed significant modulation of only the *Pycr1* gene. The reason for this difference is unclear. It is possible that the mouse intestine behaves differently than its human equivalent with regards to modulation of proline metabolism genes. Alternatively, the mouse model might reflect early adaptations in gene expression compared to the advanced cancerous lesions comprising the TCGA dataset.

To investigate the functional relevance of *PYCR1* expression in CRC, we employed siRNA technology to deplete expression of the enzyme in CRC cell lines. Unsurprisingly, validation of the specificity of two independent siRNAs showed a lack of selectivity towards the mitochondrial *PYCR* isoforms. Notwithstanding, depletion of *PYCR1* and *2* elicits cell cycle arrest and apoptosis in a variety of CRC cell lines, suggesting an important role for proline biosynthesis in CRC progression. Interestingly, the reduce cell growth in *PYCR*-depleted cells persisted when cells were grown in HPLM medium [31], providing additional support to the physiological relevance of proline metabolism for the growth of CRC cells. We also attempted to disentangle the contribution of *PYCR1* and *PYCR2* to cell growth using custom isoform-selective siRNAs. This experiment revealed that both isoforms likely contributes to CRC and unveiled an unexpected epistatic relation between the two genes, as depletion of *PYCR1* also decreased the expression of the PYCR2 protein, while the opposite was not observed. The mechanisms responsible for this regulation remain to be investigated.

The mechanisms underlying the ability of mtPYCRs to support CRC cell proliferation and survival are still unclear. External supplementation of the culture medium with either proline or nucleosides failed to restore cellular proliferation, indicating that these metabolites are not limiting in mtPYCR depleted cells. This outcome is not surprising, as proline-independent mechanisms, such as control of redox homeostasis, have been reported to account for *PYCRs* activity in cancer [4,5]. These results, together with the unaltered NADP/NADPH ratio, also argues against the involvement of the proline cycle in CRC progression. It should be noted however that others have shown that supplementation of growth medium with NADP+ is sufficient to rescue loss on *PYCR1* expression in hepatocellular carcinoma cells [17], suggesting altered redox balance following inhibition of proline biosynthesis. Yan et al. have suggested that *PYCR1* modulates p38 MAPK and NFkB signaling pathways in CRC [18]. Indeed, we observe an increase in phosphorylated p38 in RKO cells following depletion of *PYCR1*, but not in HCT116 cells (S6F Fig), suggesting that different signaling pathways are important in different CRC cell lines and outlining a heterogeneous response of CRC to manipulation of proline metabolism.

In summary, our data demonstrate that mitochondrial *PYCR* genes sustain proliferation and survival of CRC cells and suggest a pro-tumorigenic role for these genes in CRC, emphasizing the notion that proline biosynthesis is a promising target for the development of novel anti-cancer therapies [10].

## Supporting information

S1 Fig Schematic representation of the proline metabolism pathway and the proline cycle. **A)** Most of the metabolism of proline is localized in the mitochondrion, although the PYCR3 enzyme is localized in the cytoplasm. The main precursors of proline are glutamine/glutamate and ornithine. Therefore, proline metabolism is linked to the tricarboxylic acid cycle (TCA) and the urea cycle. **B)** The proline cycle enables transfer of reductive equivalent from the cytosol to the mitochondria. This exchange leads to the oxidation of NADPH, thus driving the oxidative arm of the PPP. The resulting synthesis of ribose sugar will then support nucleotide biosynthesis and DNA replication. P5CS, pyrroline 5 carboxylate synthase; P5CDH, pyrroline 5 carboxylate dehydrogenase; OAT, ornithine aminotransferase.
(TIF)

S2 Fig Expression of *PYCR* genes in human CRC. **A)** TCGA dataset analysis of the indicated proline metabolism genes in the CRC TCGA dataset (n = 41 normal and 41 cancers). Data were analyzed using unpaired *t*-test, ns = non-significant, p> 0.05, ** p<0.01, **** p<0.0001. **B)** PYCR1 H-score was assessed in a second CRC TMA. Each dot represents a single core for normal specimens and the average of two separate cores for CRC cases. Lines indicate mean ± SD and data were analyzed by two-tailed t-test (n = 16 normal and 16 cancers for each TMA). * p≤0.05. **B)** Pie chart distribution of PYCR1 protein expression in the combined TMA cohorts.
(TIF)

S3 Fig Survival analysis. Kaplan Meyer graphs showing the five-year survival rate (time shown in days) of colon adenocarcinoma patients using ’limma pipeline’ for TCGA data. Patients were separated along the median into high (UP) or low (DOWN) level of the gene of interest: **(A)** *PYCR1*, **(B)** *PYCR2* and **(C)** *PYCR3* Strata/Number at Risk indicates number of patients in each group at each time. P values were determined by log rank test and were considered statistically significant if less than 0.05. n = 434 patients. The results shown here are based upon data generated by the TCGA Research Network: http://cancergenome.nih.gov/. **D)** Cox regression analysis of stage IV colon adenocarcinoma patients in the TCGA dataset. ‘DOWN’ refers to patient samples with *PYCR2* levels lower than the median value and ‘UP’ refers to patient samples with a *PYCR2* level higher than the median value. P values were determined by log rank test.
(TIF)

S4 Fig Expression of proline metabolism enzymes in a mouse model of CRC. **A)** and **B)** mRNA levels of the indicated genes were quantified by real-time PCR in *Lgr5-Cre*^*ER*^*/Apc*^*fl/fl*^ mice at 3 days (A) and one week (B) after tamoxifen injection. The scatter blots represent the ΔCT values in *Apc* deleted mice and *Apc* WT controls. Each dot represents one mouse, and horizontal bars indicate mean ± SD. Normalization was obtained with the geometric mean value obtained from two endogenous housekeeping genes, *Pop4* and *Efnb2*. Data were analyzed using two-tailed *t*-test (n = 6 *Apc* WT controls and 6 *Apc* deleted mice). ns = p>0.05.
(TIF)

S5 Fig Knock-down of *PYCR1* decreases proliferation of CRC cell lines. **A)** SW620 cells were transfected with scramble siRNA or with *PYCR* targeting siRNA. Expression of *PYCR1*, *PYCR2* and *PYCR3* gene expression was then assessed 72 hours after transfection by real-time PCR. The graphs show the PYCR isozymes mRNA expression levels expressed as % of scramble control. The bars represent mean ± SD. One-way ANOVA with Tukey’s multiple comparisons test (n = 3 independent experiments). *** p<0.001, **** p<0.0001, ns indicates no significant change. **B)** Bar graphs showing number of SW620 cells 72 hours after transfection with siRNAs targeting *PYCR* (si-mtPYCRs) and control siRNA (Scr). Untr indicates control cells with no treatment. The bars represent mean ± SD. Data were analyzed using One-way ANOVA and Tukey’s multiple comparisons test (n = 3 independent experiments). *** p<0.001, **** p<0.0001. **C)** Bar graphs showing number of RKO cells grown in HPLM and transfected for 72 hours with siRNAs targeting *PYCR* (si-mtPYCRs) and control siRNA (Scr). The bars represent mean ± SD. Data were analyzed using two-tailed t-test (n = 3 technical replicates). * p≤0.05. The accompanying western blots confirm the reduction in PYCR1 protein and increased p21 protein in siRNA transfected HCEC cells. Actin was used as loading control. **D)** Bar graphs showing number of HCEC 72 hours after transfection with siRNAs targeting *PYCR* (si-mtPYCRs) and control siRNA (Scr). Untr indicates control cells with no treatment. The bars represent mean ± SD. Data were analyzed using one-way ANOVA and Tukey’s multiple comparisons test (n = 3 independent experiments). ns = p>0.05. The accompanying western blots confirm the reduction in PYCR1 protein in siRNA transfected HCEC cells. Actin was used as loading control.
(TIF)

S6 Fig Knock-down of *PYCR* genes effect is independent of proline and nucleotide biosynthesis. **A)** Representative western blot showing downregulation of PYCR1 protein in RKO cells 24 and 48 hours after transfection for analysis by mass-spectrometry. **B)** Intracellular proline levels in RKO cells measured using ninhydrine reaction. Cells were transfected as indicated for 72 hours in the presence of physiological proline concentration or supplemented with 5 mM proline. The bars represent mean ± SD. **C)** Representative Western blots of experiments performed at least in duplicate, showing lack of change in the mTOR and AAR pathways in HCT116 cells. Tubulin and actin were used as loading control and nutrient starved cells were used as positive control. **D)** Bar graphs showing number of RKO cells 72 hours after transfection with siRNAs targeting *PYCR* genes (si-mtPYCRs) and control siRNA (Scr) or left untreated (Untr). Cell were supplemented with vehicle or nucleosides as indicated. The bars represent mean ± SD. Data were analyzed using two-way ANOVA and Sidak’s multiple comparisons test (n = 3 independent experiments). * p≤0.05. **E)** Graph showing unaltered NADPH/NADP ratio in cells transfect with scr or siRNA targeting mtPYCRs. The bars represent mean ± SD. Data were analyzed using *t*-test (scr vs si-mtPYCRs, n = 3 independent experiments). ns = p>0.05. **F)** Western blots show an increased phosphorylation of p38 in RKO cells and no change in HCT116 cells following a 72-hour knockdown of *PYCR1*. Tubulin was used as loading control and nutrient starved cells were used as positive control for p38 phosphorylation.
(TIF)

S1 Table List of antibodies used for western blotting. (DOCX)

S2 Table List of TaqMan rtPCR essays. Mouse Actd, Tbp, Ywhz, Gusb, Pop4, Efnb2, Gapdh, Hprt, B2m were used for NormFinder analysis of candidate endogenous controls.
(DOCX)

S1 Raw images (PDF)
