# Supplementary figures and images for: Increased mitochondrial proline metabolism sustains proliferation and survival of colorectal cancer cells
Source: PLoS One. 2022 Feb 7;17(2):e0262364. doi: 10.1371/journal.pone.0262364 (PMC8820619; doi:10.1371/journal.pone.0262364)

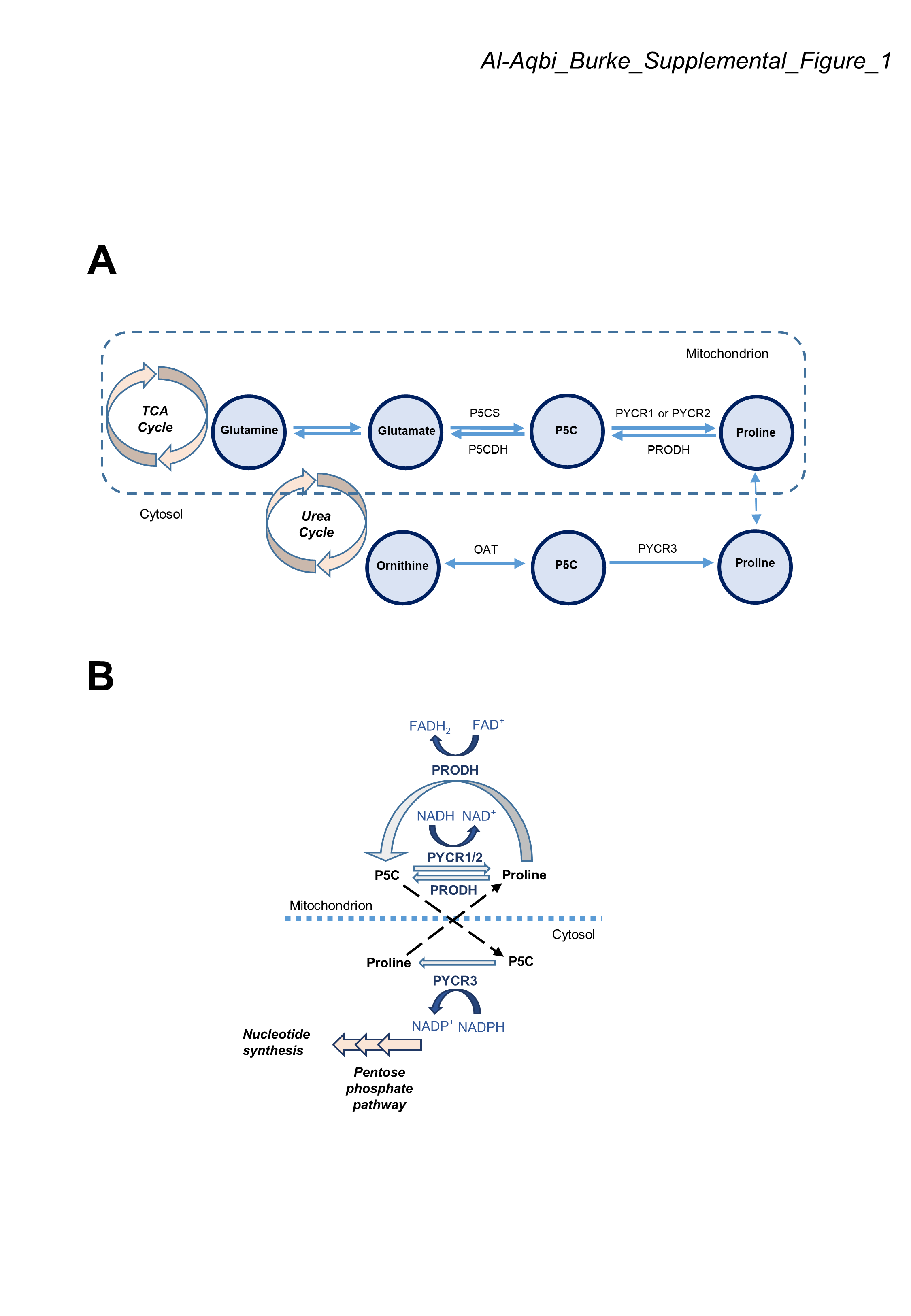

Supplement: S1 Fig — A) Most of the metabolism of proline is localized in the mitochondrion, although the PYCR3 enzyme is localized in the cytoplasm. The main precursors of proline are glutamine/glutamate and ornithine. Therefore, proline metabolism is linked to the tricarboxylic acid cycle (TCA) and the urea cycle. B) The proline cycle enables transfer of reductive equivalent from the cytosol to the mitochondria. This exchange leads to the oxidation of NADPH, thus driving the oxidative arm of the PPP. The resulting synthesis of ribose sugar will then support nucleotide biosynthesis and DNA replication. P5CS, pyrroline 5 carboxylate synthase; P5CDH, pyrroline 5 carboxylate dehydrogenase; OAT, ornithine aminotransferase. (TIF) [file pone.0262364.s001.TIF]

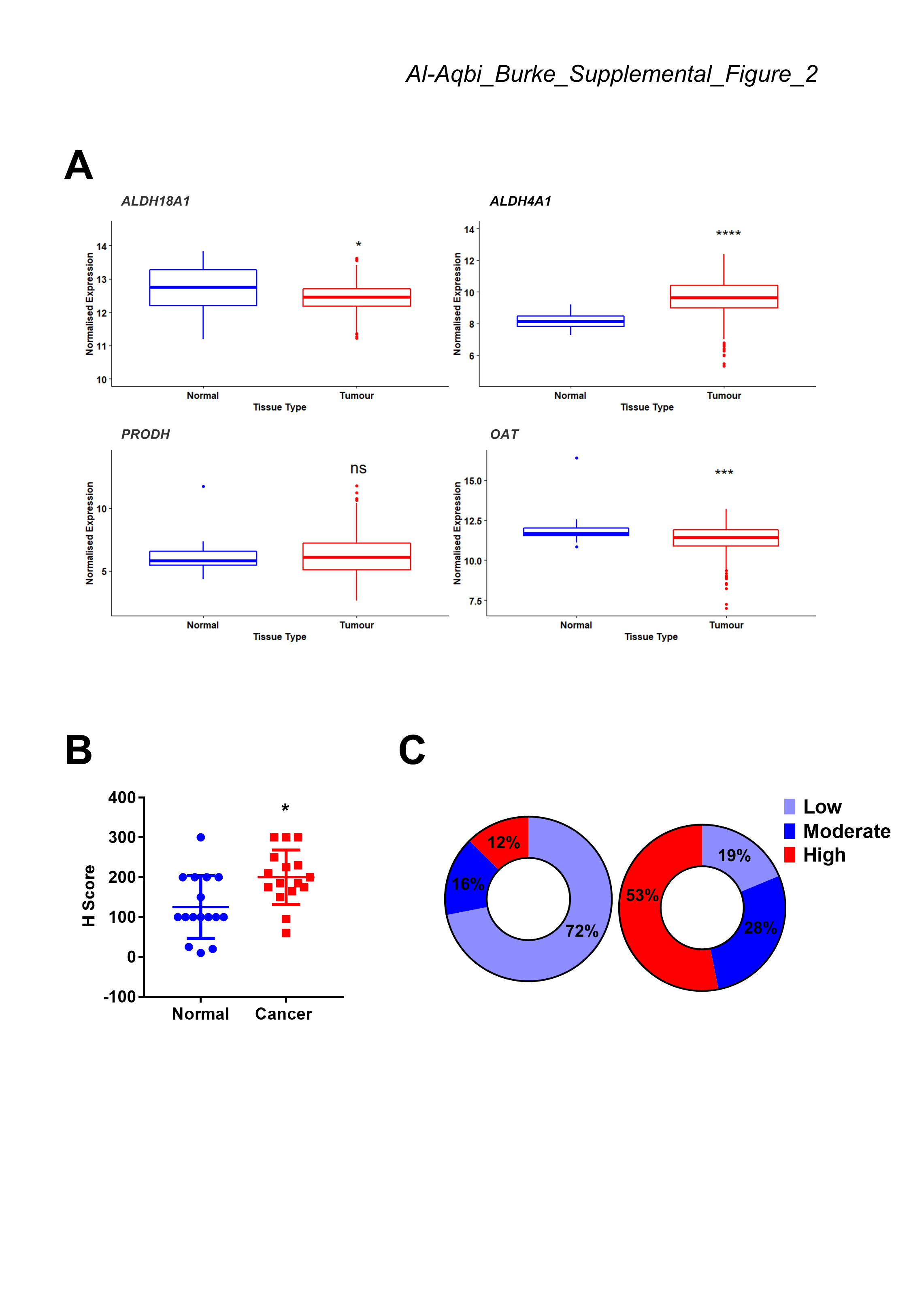

Supplement: S2 Fig — A) TCGA dataset analysis of the indicated proline metabolism genes in the CRC TCGA dataset (n = 41 normal and 41 cancers). Data were analyzed using unpaired t-test, ns = non-significant, p> 0.05, ** p<0.01, **** p<0.0001. B) PYCR1 H-score was assessed in a second CRC TMA. Each dot represents a single core for normal specimens and the average of two separate cores for CRC cases. Lines indicate mean ± SD and data were analyzed by two-tailed t-test (n = 16 normal and 16 cancers for each TMA). * p≤0.05. B) Pie chart distribution of PYCR1 protein expression in the combined TMA cohorts. (TIF) [file pone.0262364.s002.TIF]

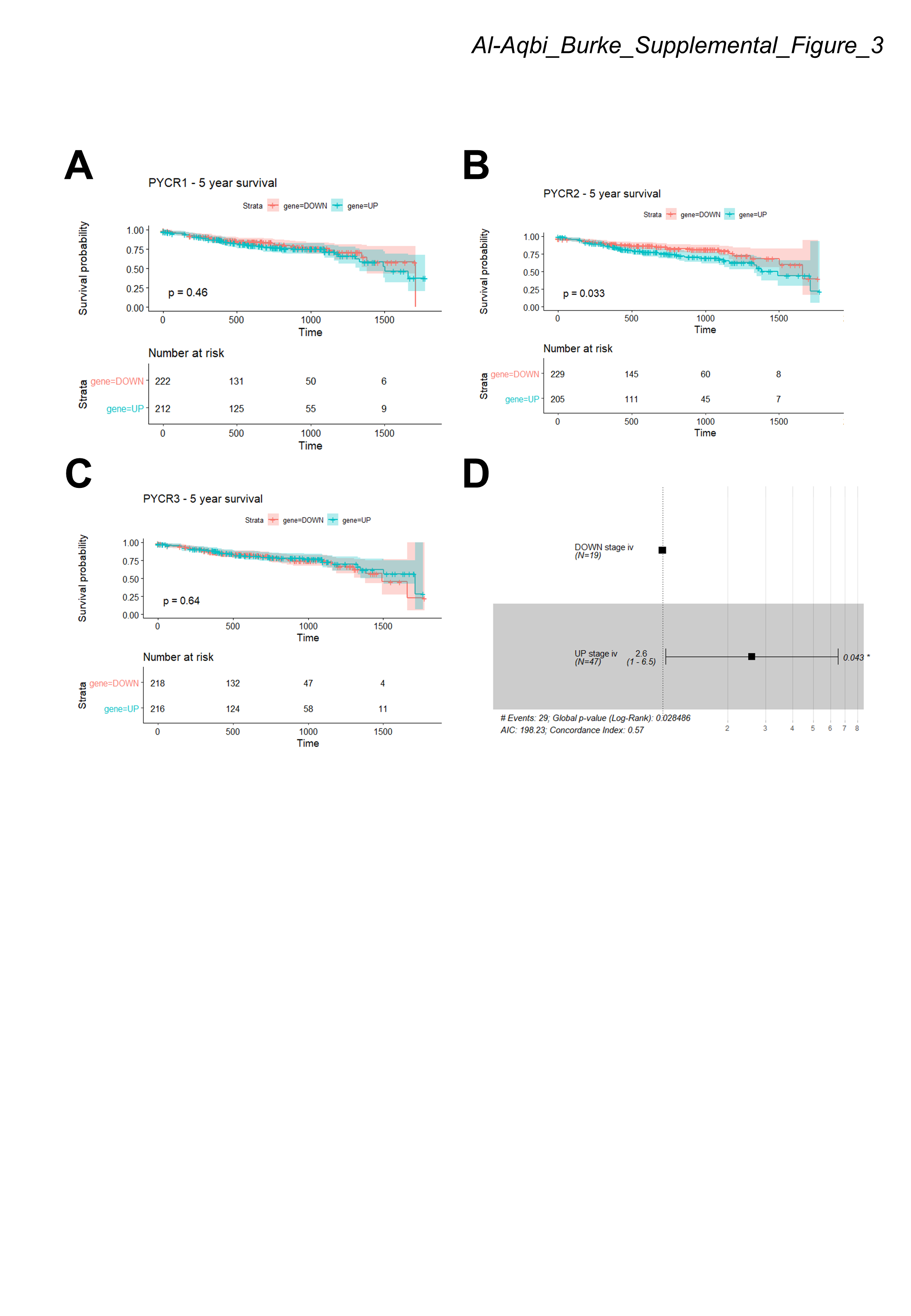

Supplement: S3 Fig — Kaplan Meyer graphs showing the five-year survival rate (time shown in days) of colon adenocarcinoma patients using ’limma pipeline’ for TCGA data. Patients were separated along the median into high (UP) or low (DOWN) level of the gene of interest: (A) PYCR1, (B) PYCR2 and (C) PYCR3 Strata/Number at Risk indicates number of patients in each group at each time. P values were determined by log rank test and were considered statistically significant if less than 0.05. n = 434 patients. The results shown here are based upon data generated by the TCGA Research Network: http://cancergenome.nih.gov/. D) Cox regression analysis of stage IV colon adenocarcinoma patients in the TCGA dataset. ‘DOWN’ refers to patient samples with PYCR2 levels lower than the median value and ‘UP’ refers to patient samples with a PYCR2 level higher than the median value. P values were determined by log rank test. (TIF) [file pone.0262364.s003.TIF]

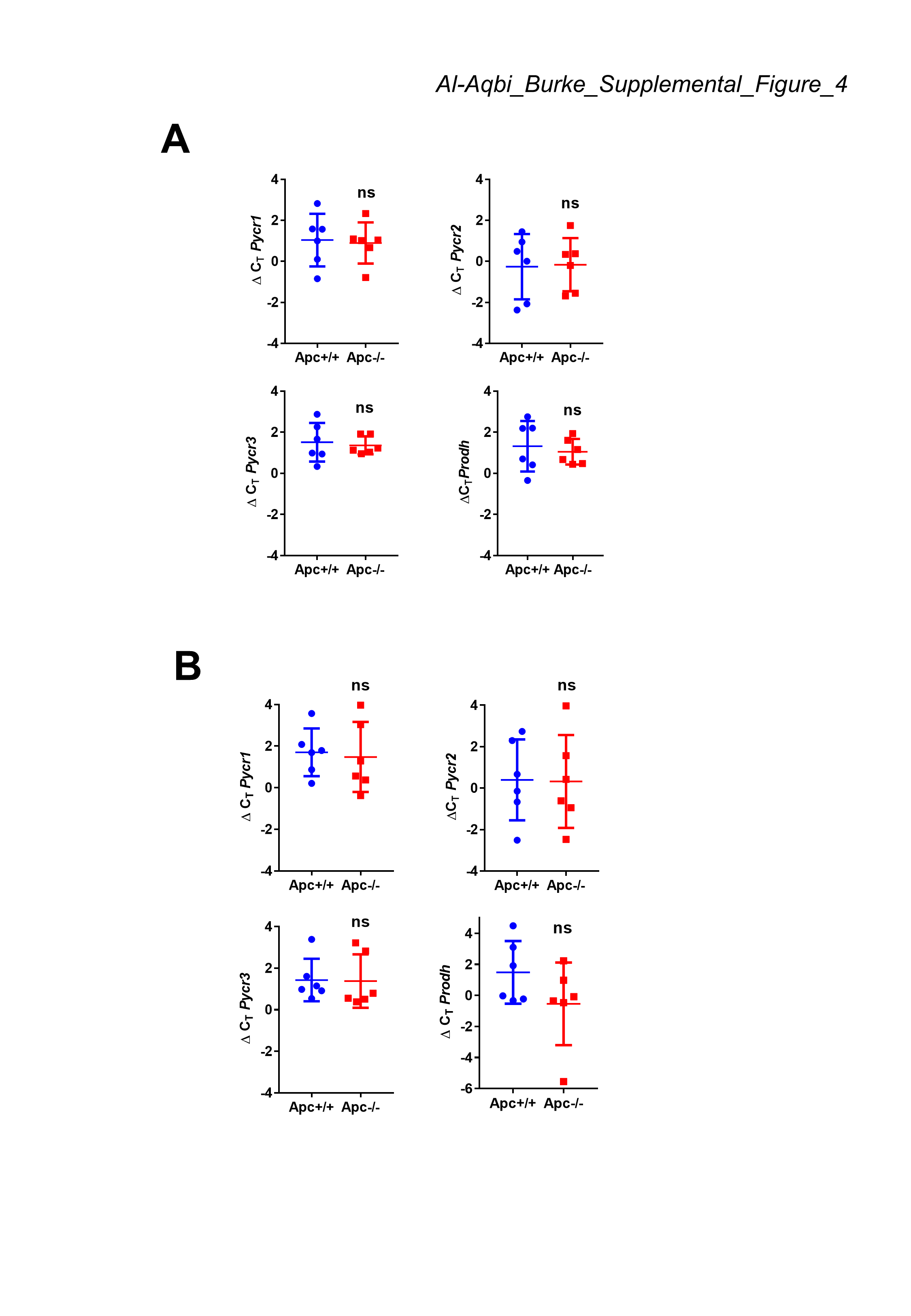

Supplement: S4 Fig — A) and B) mRNA levels of the indicated genes were quantified by real-time PCR in Lgr5-CreER/Apcfl/fl mice at 3 days (A) and one week (B) after tamoxifen injection. The scatter blots represent the ΔCT values in Apc deleted mice and Apc WT controls. Each dot represents one mouse, and horizontal bars indicate mean ± SD. Normalization was obtained with the geometric mean value obtained from two endogenous housekeeping genes, Pop4 and Efnb2. Data were analyzed using two-tailed t-test (n = 6 Apc WT controls and 6 Apc deleted mice). ns = p>0.05. (TIF) [file pone.0262364.s004.TIF]

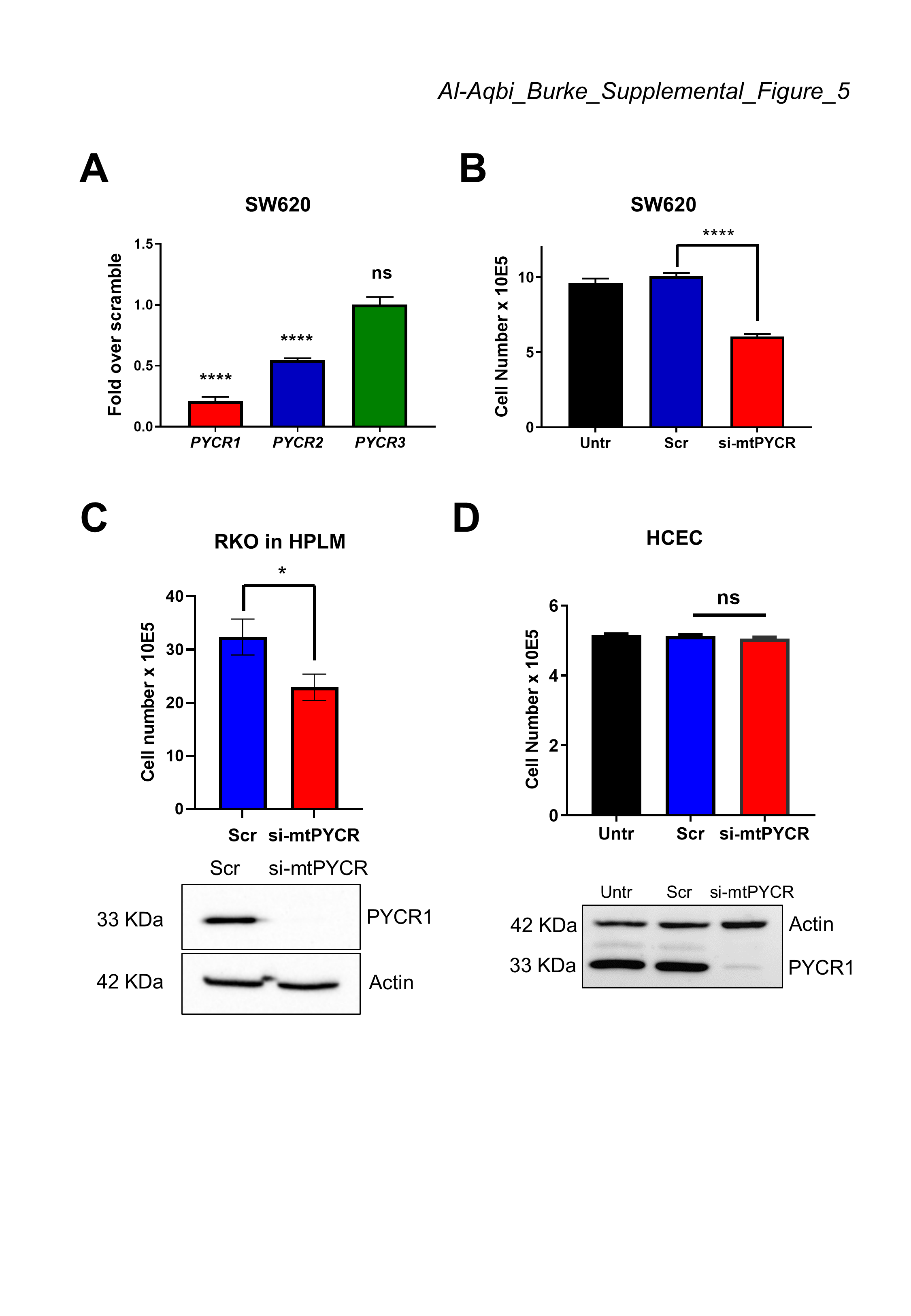

Supplement: S5 Fig — A) SW620 cells were transfected with scramble siRNA or with PYCR targeting siRNA. Expression of PYCR1, PYCR2 and PYCR3 gene expression was then assessed 72 hours after transfection by real-time PCR. The graphs show the PYCR isozymes mRNA expression levels expressed as % of scramble control. The bars represent mean ± SD. One-way ANOVA with Tukey’s multiple comparisons test (n = 3 independent experiments). *** p<0.001, **** p<0.0001, ns indicates no significant change. B) Bar graphs showing number of SW620 cells 72 hours after transfection with siRNAs targeting PYCR (si-mtPYCRs) and control siRNA (Scr). Untr indicates control cells with no treatment. The bars represent mean ± SD. Data were analyzed using One-way ANOVA and Tukey’s multiple comparisons test (n = 3 independent experiments). *** p<0.001, **** p<0.0001. C) Bar graphs showing number of RKO cells grown in HPLM and transfected for 72 hours with siRNAs targeting PYCR (si-mtPYCRs) and control siRNA (Scr). The bars represent mean ± SD. Data were analyzed using two-tailed t-test (n = 3 technical replicates). * p≤0.05. The accompanying western blots confirm the reduction in PYCR1 protein and increased p21 protein in siRNA transfected HCEC cells. Actin was used as loading control. D) Bar graphs showing number of HCEC 72 hours after transfection with siRNAs targeting PYCR (si-mtPYCRs) and control siRNA (Scr). Untr indicates control cells with no treatment. The bars represent mean ± SD. Data were analyzed using one-way ANOVA and Tukey’s multiple comparisons test (n = 3 independent experiments). ns = p>0.05. The accompanying western blots confirm the reduction in PYCR1 protein in siRNA transfected HCEC cells. Actin was used as loading control. (TIF) [file pone.0262364.s005.TIF]

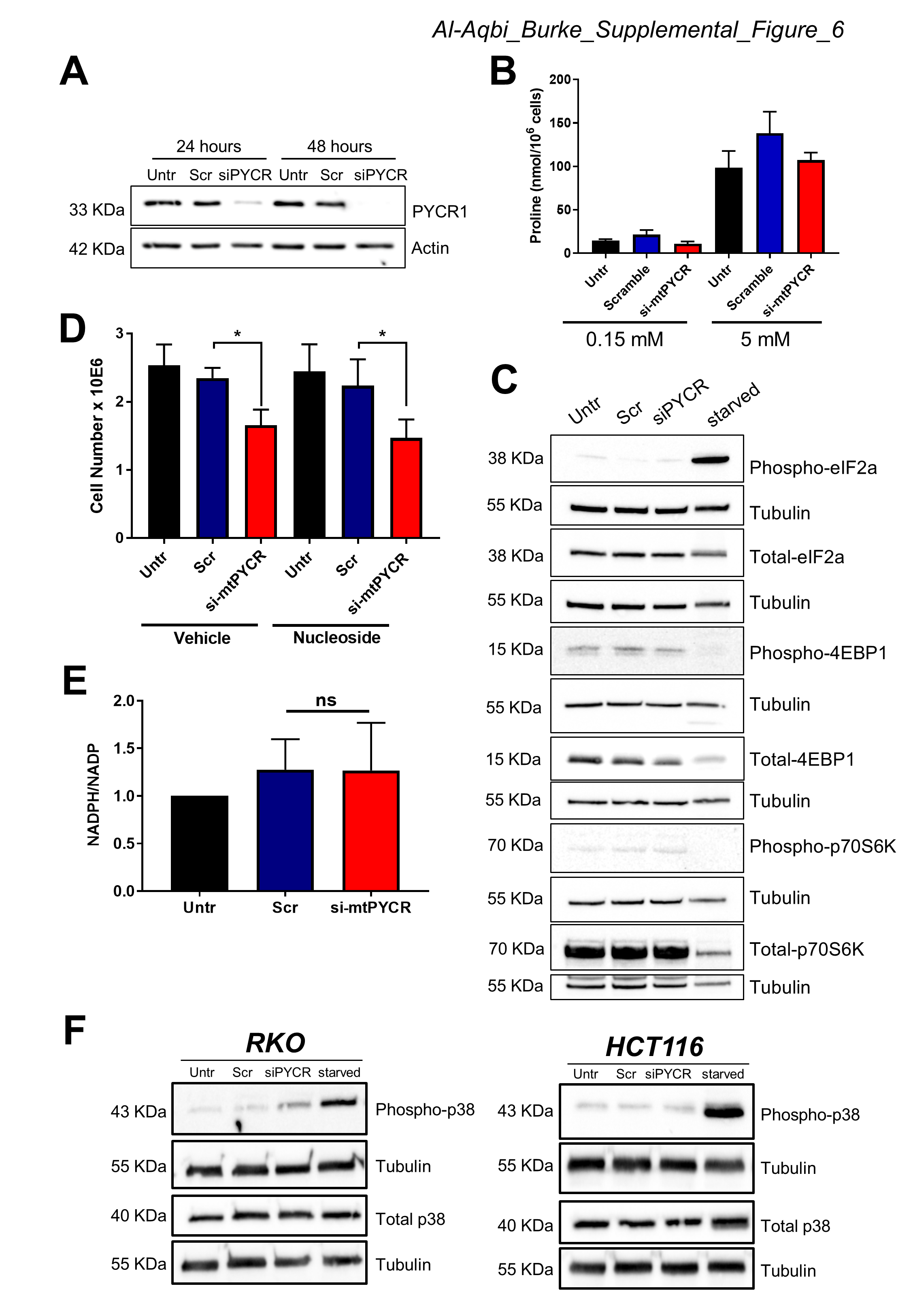

Supplement: S6 Fig — A) Representative western blot showing downregulation of PYCR1 protein in RKO cells 24 and 48 hours after transfection for analysis by mass-spectrometry. B) Intracellular proline levels in RKO cells measured using ninhydrine reaction. Cells were transfected as indicated for 72 hours in the presence of physiological proline concentration or supplemented with 5 mM proline. The bars represent mean ± SD. C) Representative Western blots of experiments performed at least in duplicate, showing lack of change in the mTOR and AAR pathways in HCT116 cells. Tubulin and actin were used as loading control and nutrient starved cells were used as positive control. D) Bar graphs showing number of RKO cells 72 hours after transfection with siRNAs targeting PYCR genes (si-mtPYCRs) and control siRNA (Scr) or left untreated (Untr). Cell were supplemented with vehicle or nucleosides as indicated. The bars represent mean ± SD. Data were analyzed using two-way ANOVA and Sidak’s multiple comparisons test (n = 3 independent experiments). * p≤0.05. E) Graph showing unaltered NADPH/NADP ratio in cells transfect with scr or siRNA targeting mtPYCRs. The bars represent mean ± SD. Data were analyzed using t-test (scr vs si-mtPYCRs, n = 3 independent experiments). ns = p>0.05. F) Western blots show an increased phosphorylation of p38 in RKO cells and no change in HCT116 cells following a 72-hour knockdown of PYCR1. Tubulin was used as loading control and nutrient starved cells were used as positive control for p38 phosphorylation. (TIF) [file pone.0262364.s006.TIF]
