# Supplementary material for: Increased mitochondrial proline metabolism sustains proliferation and survival of colorectal cancer cells
Source: PLoS One. 2022 Feb 7;17(2):e0262364. doi: 10.1371/journal.pone.0262364 (PMC8820619; doi:10.1371/journal.pone.0262364)
Supplement: S1 Table — (DOCX) [file pone.0262364.s007.docx]

| Antibody | Cat# | Dilution | Host | Supplier |
| --- | --- | --- | --- | --- |
| PYCR1 | PA5-26890 | 1:1,000 | Rabbit | Thermo Fischer |
| PYCR2 | HPA056873 | 1:1,000 | Rabbit | Atlas |
| p21 | 2947 | 1:1,000 | Rabbit | Cell Signaling |
| Cyclin D1 | 2978 | 1:1,000 | Rabbit | Cell Signaling |
| Cyclin D3 | 2936 | 1:1,000 | Mouse | Cell Signaling |
| p38 | 9212 | 1:1,000 | Rabbit | Cell Signaling |
| Phospho-p38 | 9211 | 1:1,000 | Rabbit | Cell Signaling |
| EIF2A | 5324 | 1:500 | Rabbit | Cell Signaling |
| Phospho-EIF2Α | 3398 | 1:500 | Rabbit | Cell Signaling |
| 4EBP1 | 9644 | 1:10,000 | Rabbit | Cell Signaling |
| Phopsho-4EBP1 | 9451 | 1:1,000 | Rabbit | Cell Signaling |
| P70S6K | 2708 | 1:1,000 | Rabbit | Cell Signaling |
| Phospho-p70S6K | 9234 | 1:1,000 | Rabbit | Cell Signaling |
| Cleaved caspase 3 | 9664 | 1:1,000 | Rabbit | Cell Signaling |
| Cleaved PARP | 5625 | 1:1,000 | Rabbit | Cell Signaling |
| PUMA | 98672 | 1:1,000 | Rabbit | Cell Signaling |
| BAX | 89477 | 1:1,000 | Mouse | Cell Signaling |
| BAK | 12105 | 1:1,000 | Rabbit | Cell Signaling |
| BCL-2 | M0887 | 1:1,000 | Mouse | Agilent, DAKO |
| Actin | SC-1616 | 1:5,000 | Goat | Santa Cruz |
| Tubulin | T5168 | 1:5,000 | Mouse | Sigma |
| Donkey anti-goat | SC-2020 | 1:10,000 | Donkey | Santa Cruz |
| Goat anti-rabbit | SC-2030 | 1:10,000 | Goat | Santa Cruz |
| Goat anti-mouse | SC-2031 | 1:10,000 | Goat | Santa Cruz |

**S1 Table.**
