# Supplementary material for: Increased mitochondrial proline metabolism sustains proliferation and survival of colorectal cancer cells
Source: PLoS One. 2022 Feb 7;17(2):e0262364. doi: 10.1371/journal.pone.0262364 (PMC8820619; doi:10.1371/journal.pone.0262364)
Supplement: S2 Table — Mouse Actd, Tbp, Ywhz, Gusb, Pop4, Efnb2, Gapdh, Hprt, B2m were used for NormFinder analysis of candidate endogenous controls. (DOCX) [file pone.0262364.s008.docx]

| Gene | Fluorophore | Amplicon Length | Assay ID/Cat# |
| --- | --- | --- | --- |
| Human *PYCR1* | FAM | 128 | [Hs01048019_mL](http://www.thermofisher.com/taqman-gene-expression/product/Hs01048019_m1?CID=&ICID=&subtype=ge_all)/ 4351372 |
| Human *PYCR2* | FAM | 107 | [Hs00371953_mL](https://www.thermofisher.com/taqman-gene-expression/product/Hs00371953_m1?CID=&ICID=&subtype=)/ 4331182 |
| Human *PYCR3* | FAM | 110 | [Hs00225031_mL](https://www.thermofisher.com/taqman-gene-expression/product/Hs00225031_m1?CID=&ICID=&subtype=)/ 4351372 |
| Human *PRODH* | FAM | 136 | Hs00275205_mL/ 433118 |
| Human *ACTB* | VIC | 171 | Hs99999903_m1/ 4448484 |
| Mouse *Pycr1* | FAM | 82 | Mm00522674_m1/ 4331182 |
| Mouse *Pycr2* | FAM | 58 | Mm00505074_m1/ 4331182 |
| Mouse *Pycr3* | FAM | 68 | Mm01205845_m1/ 4331182 |
| Mouse *Prodh* | FAM | 76 | Mm00448398_m1/ 4351372 |
| Mouse *Actb* | VIC | 143 | Mm02619580_g1/ 4448484 |
| Mouse *Tbp* | VIC | 121 | Mm01279310_m1/ 4448484 |
| Mouse *Ywhz* | VIC | 70 | Mm01722325_m1/ 4448484 |
| Mouse *Gusb* | VIC | 93 | Mm00446957_m1/ 4448484 |
| Mouse *Pop4* | VIC | 116 | [Mm00546481_m1](https://www.thermofisher.com/taqman-gene-expression/product/Mm00546481_m1?CID=&ICID=&subtype=)/ 4448484 |
| Mouse *Efnb2* | VIC | 136 | Mm01215896_m1/ 4448484 |
| Mouse *Gapdh* | VIC | 109 | Mm99999915_g1/ 4448484 |
| Mouse *Hprt* | VIC | 131 | Mm03024075_m1/ 4448484 |
| Mouse *B2m* | VIC | 125 | Mm00437764_m1/ 4448484 |

**S2 Table.**
