# Supplementary material for: Increased mitochondrial proline metabolism sustains proliferation and survival of colorectal cancer cells
Source: PLoS One. 2022 Feb 7;17(2):e0262364. doi: 10.1371/journal.pone.0262364 (PMC8820619; doi:10.1371/journal.pone.0262364)
Supplement: S1 Raw images — (PDF) [file pone.0262364.s009.pdf]

Original western blot images used in figures

FIGURE 1C

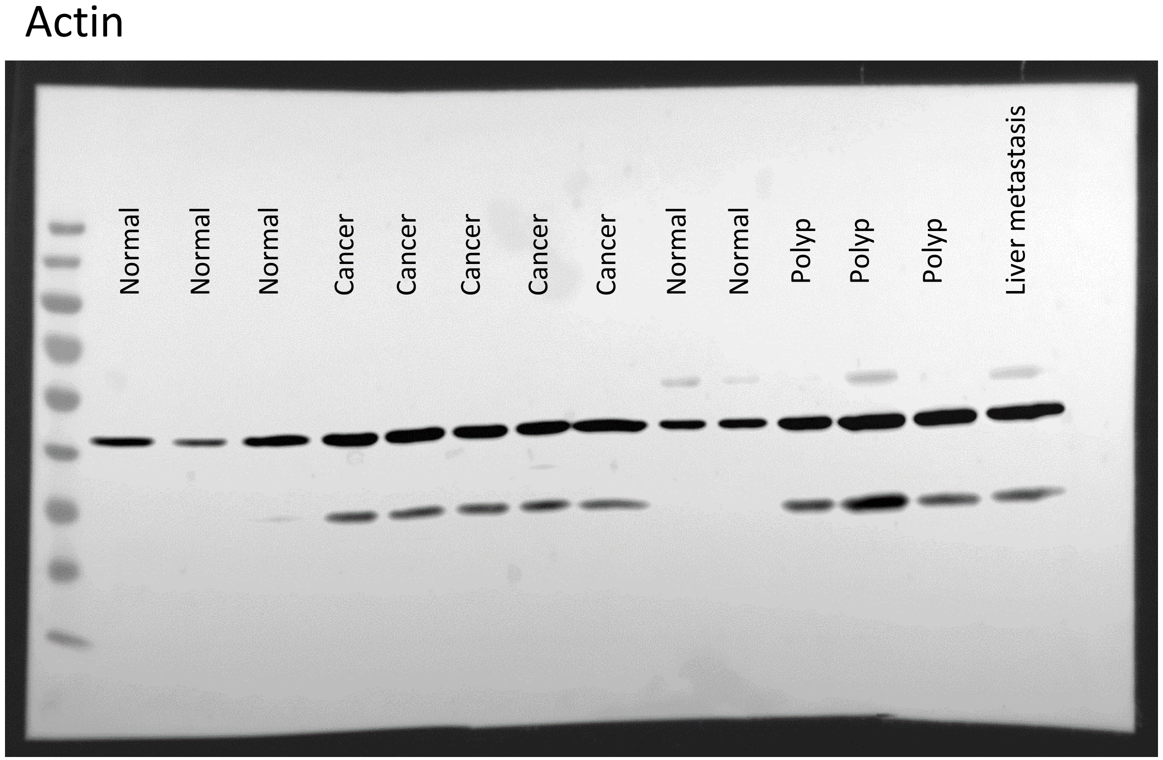

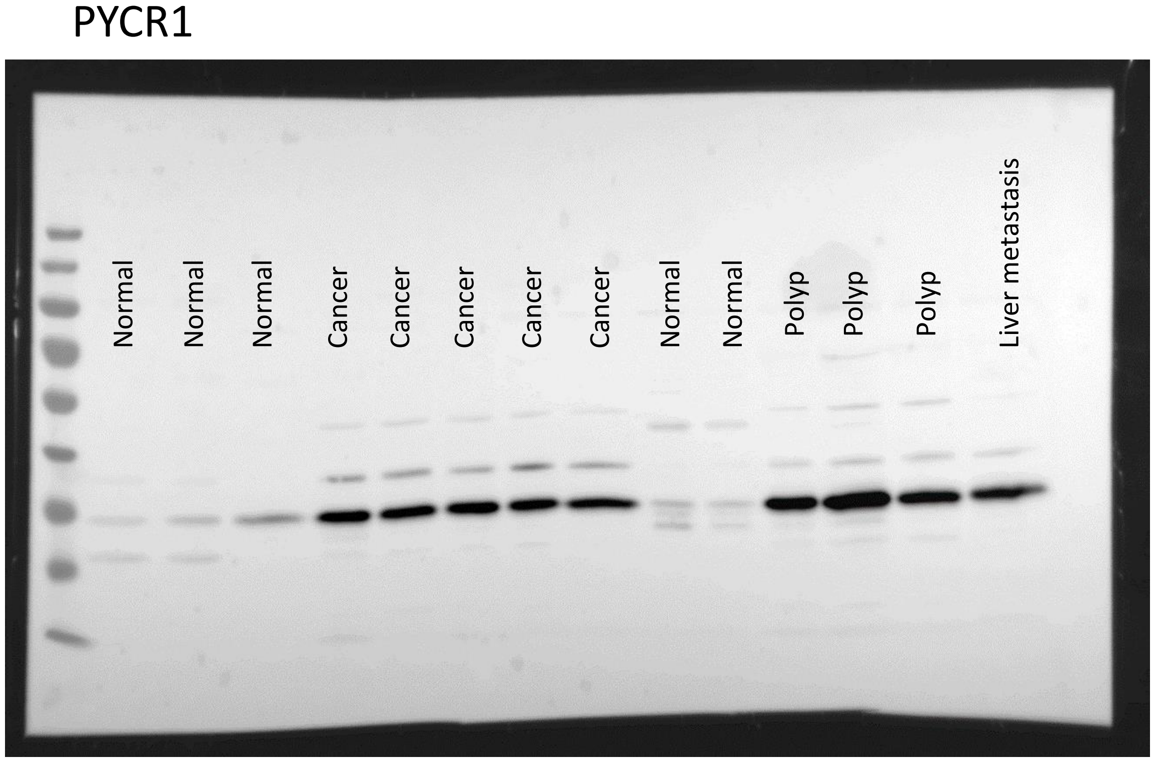

Original western blot images used in figures

FIGURE 1D

Actin

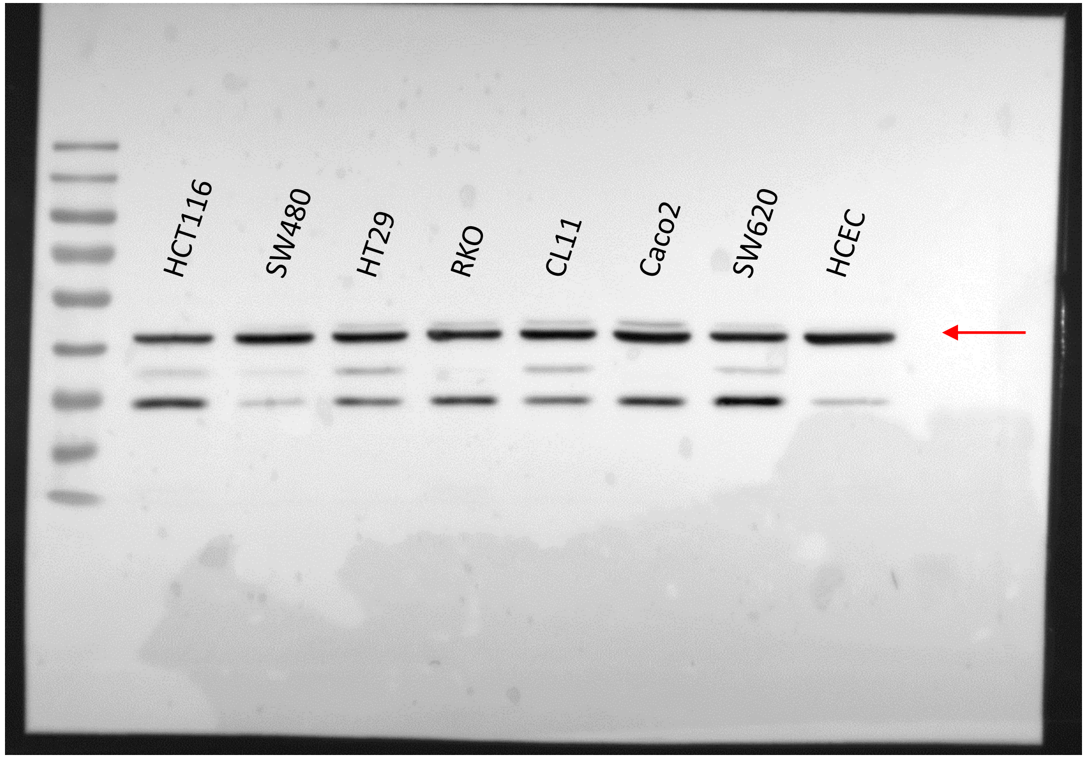

PYCR1

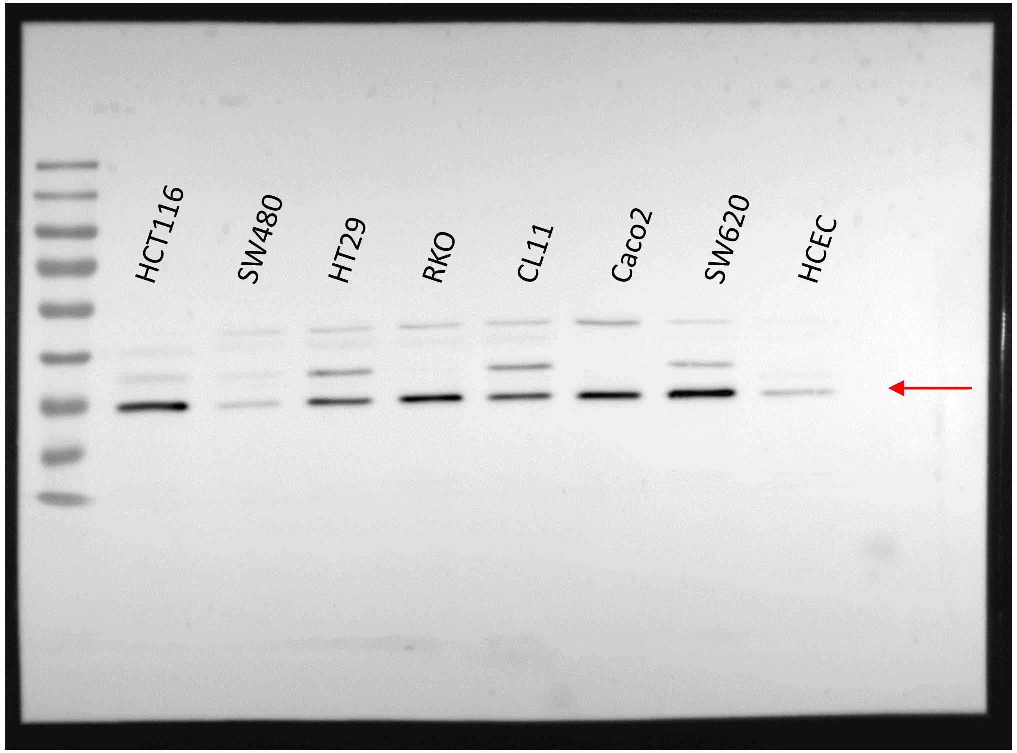

Original western blot images used in figures

FIGURE 1D

Actin

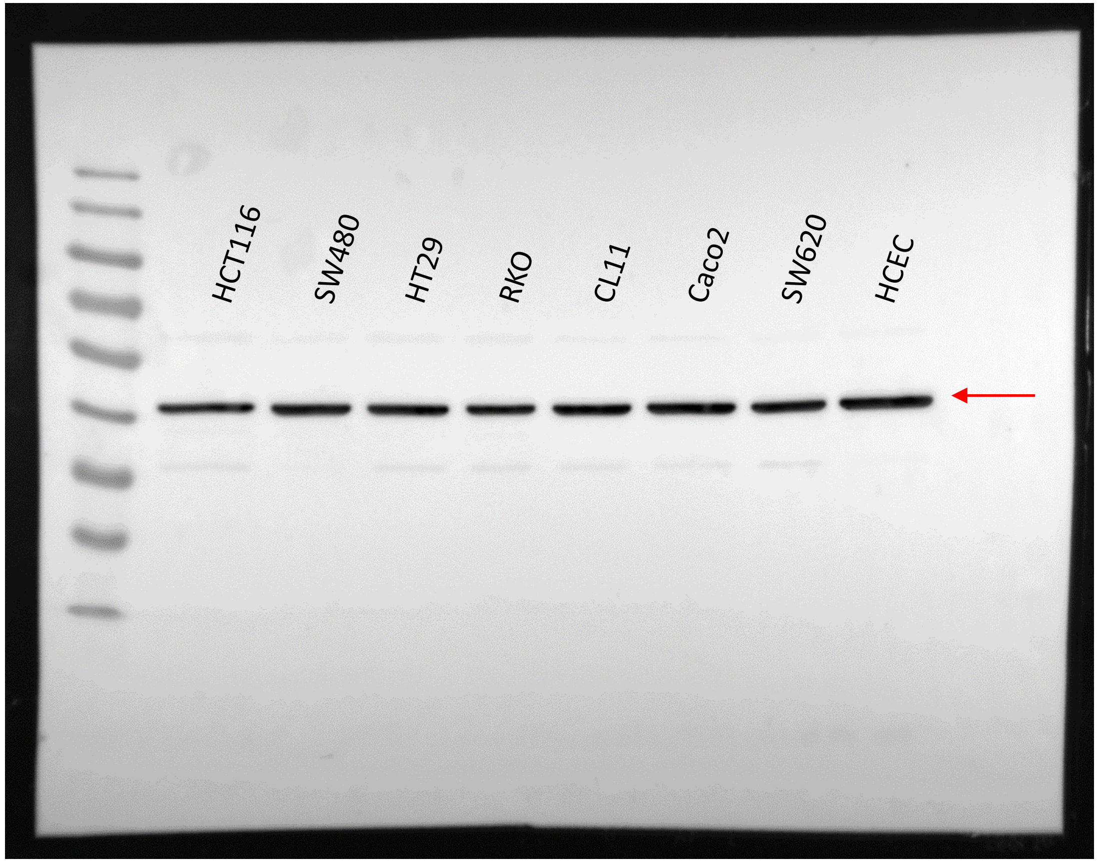

PYCR2

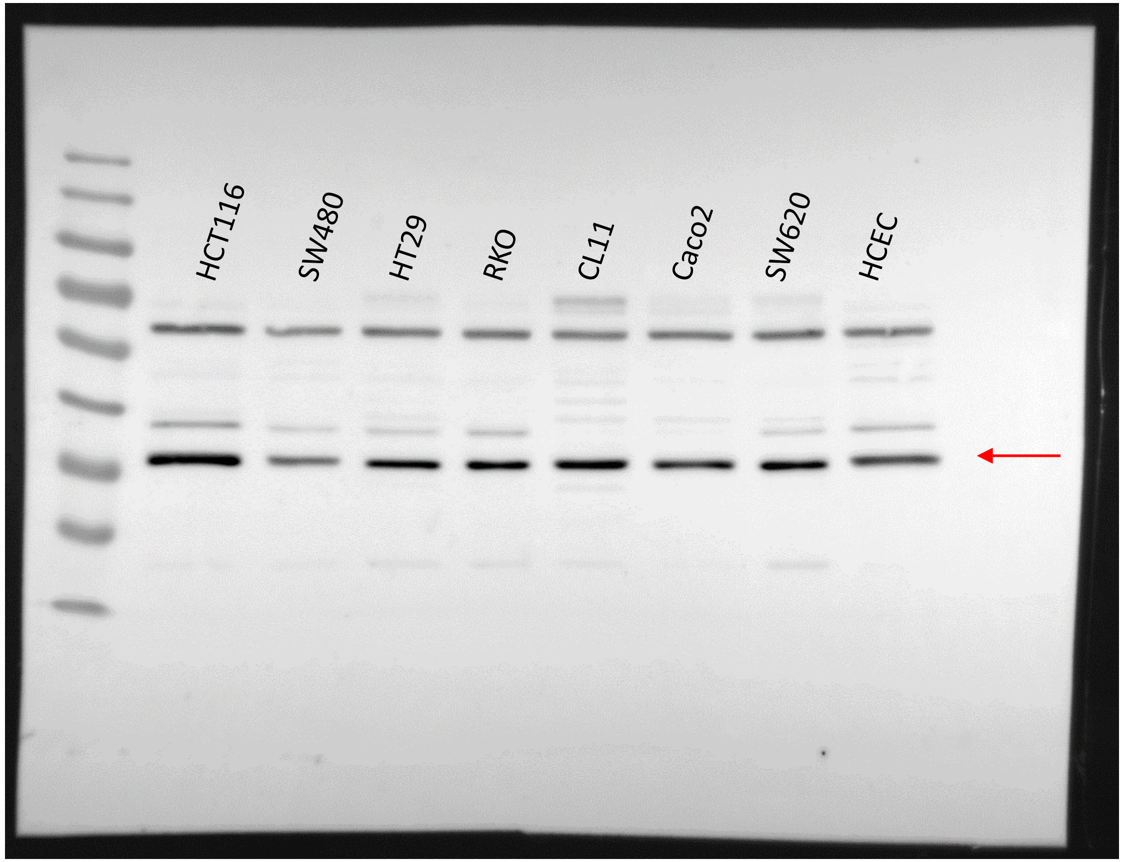

Original western blot images used in figures

FIGURE 3

RKO

HCT116

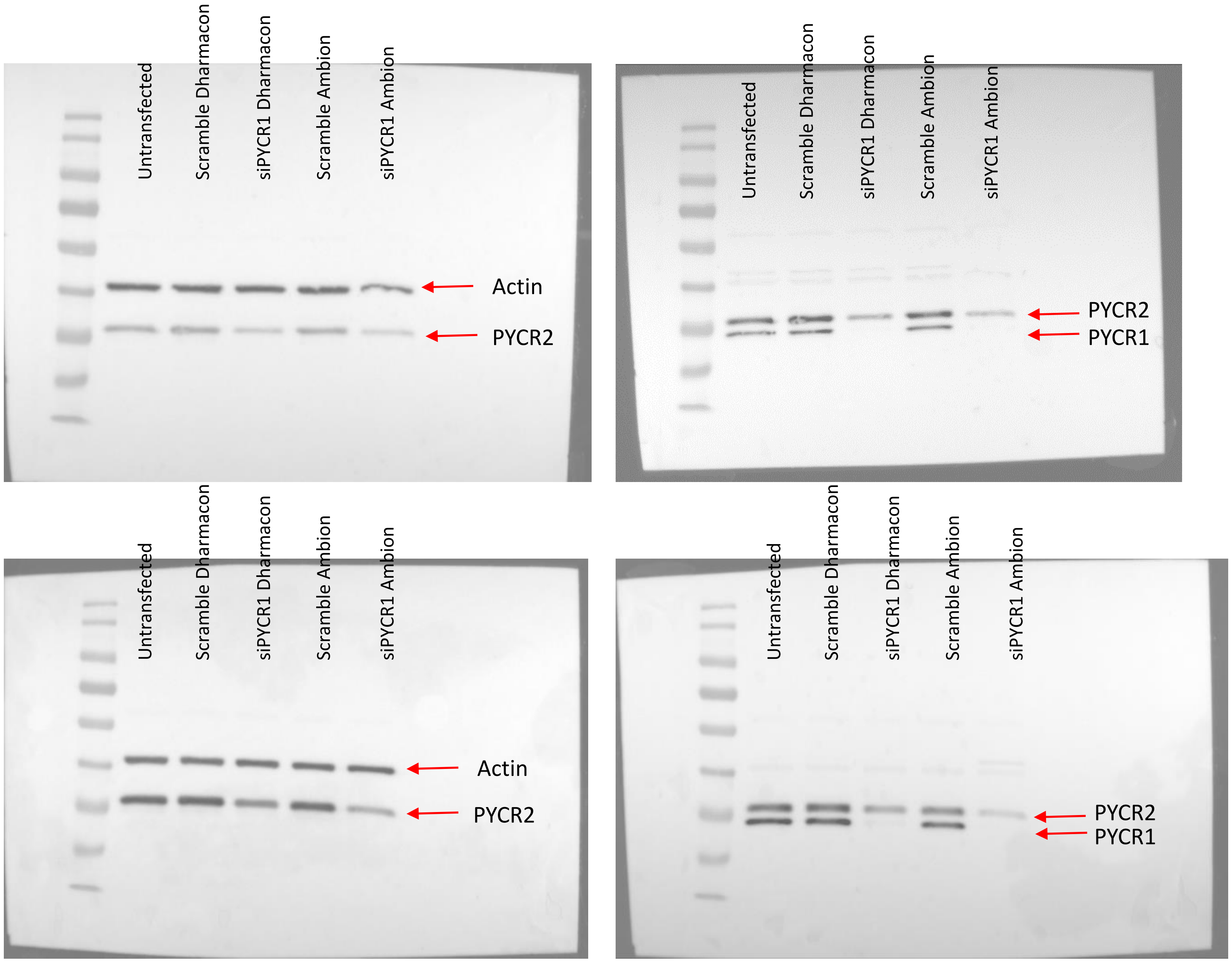

Original western blot images used in figures

FIGURE 4D

RKO cells

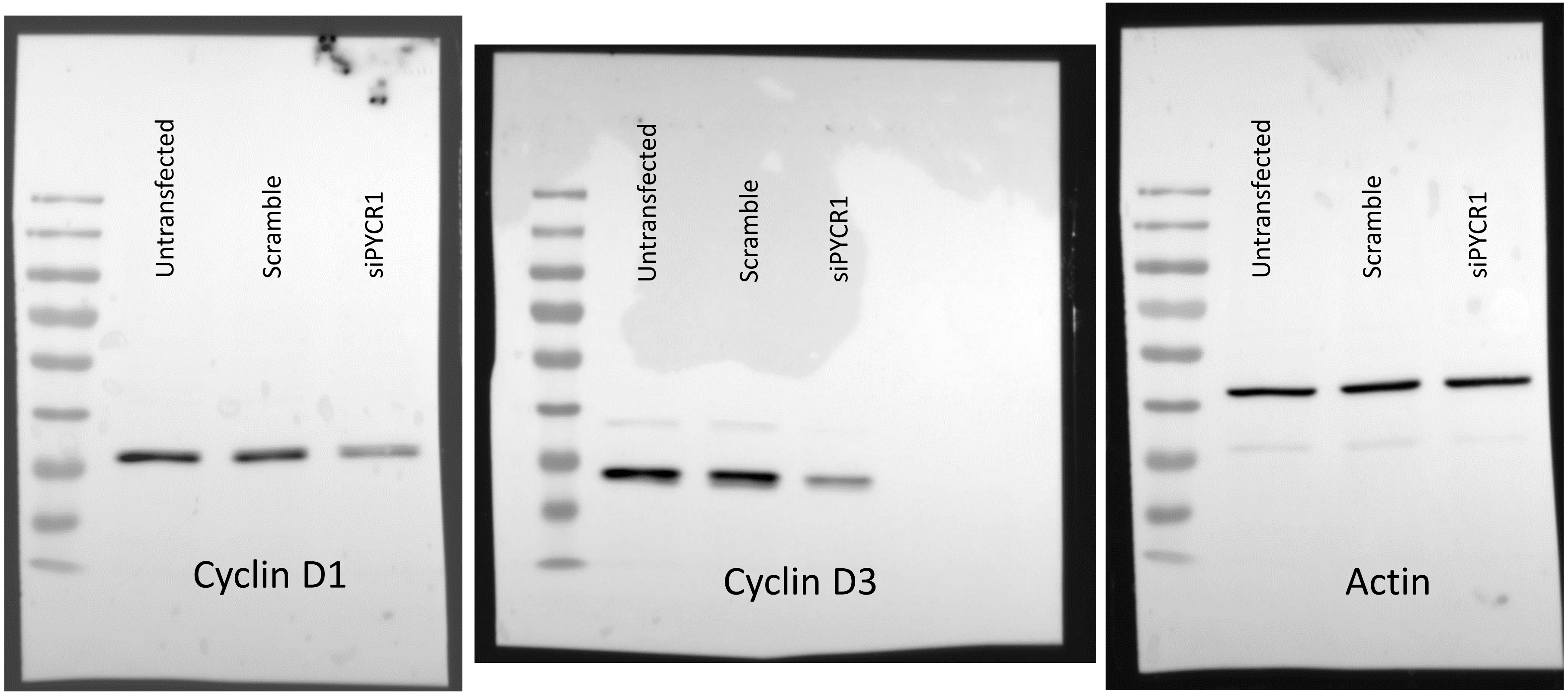

Original western blot images used in figures

FIGURE 4D

HCT116 cells

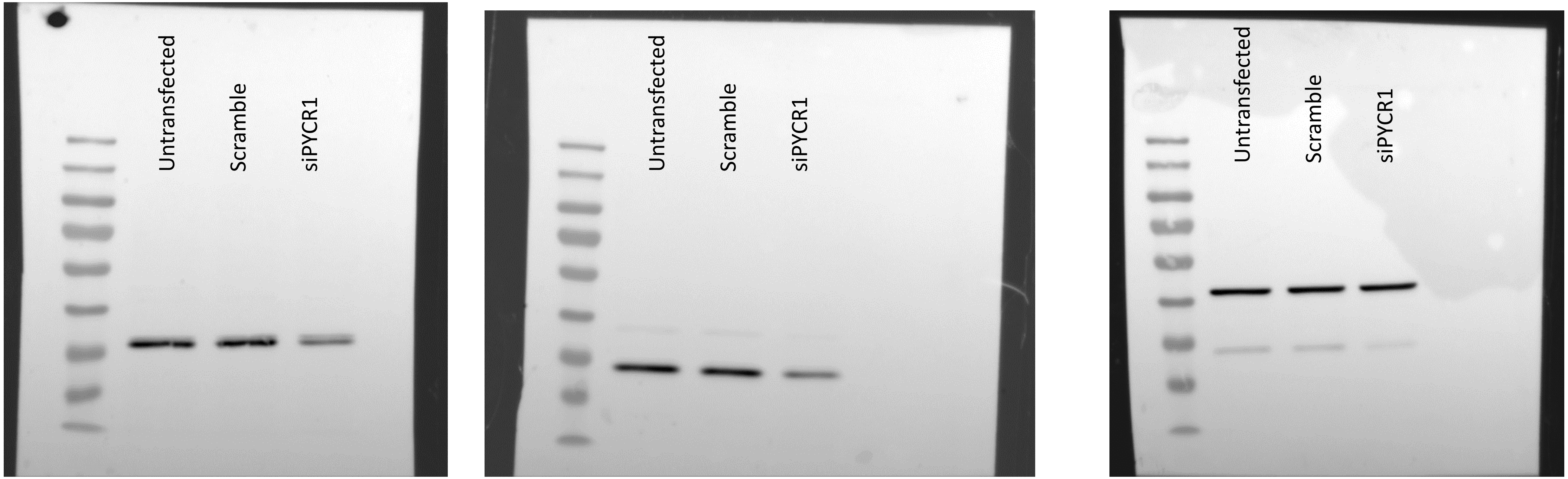

Original western blot images used in figures

FIGURE 4D

SW620 cells

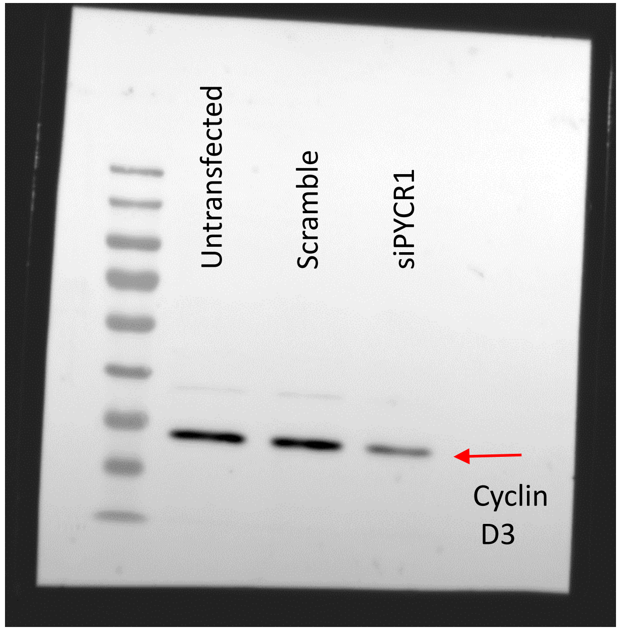

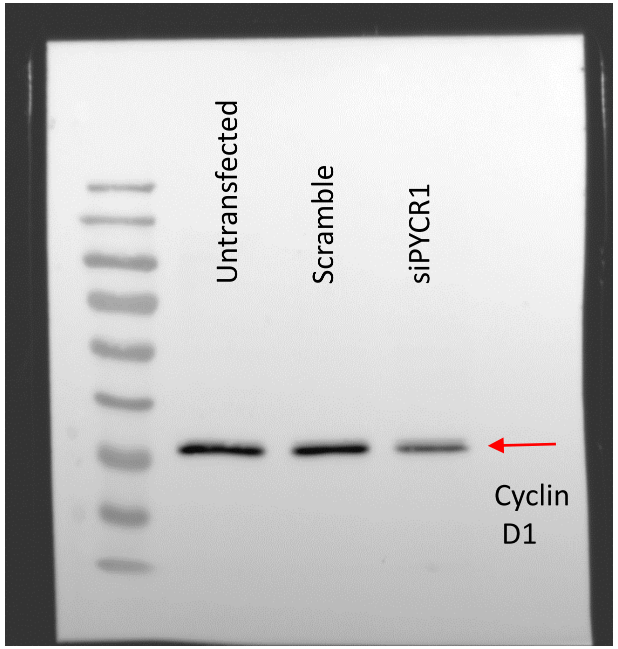

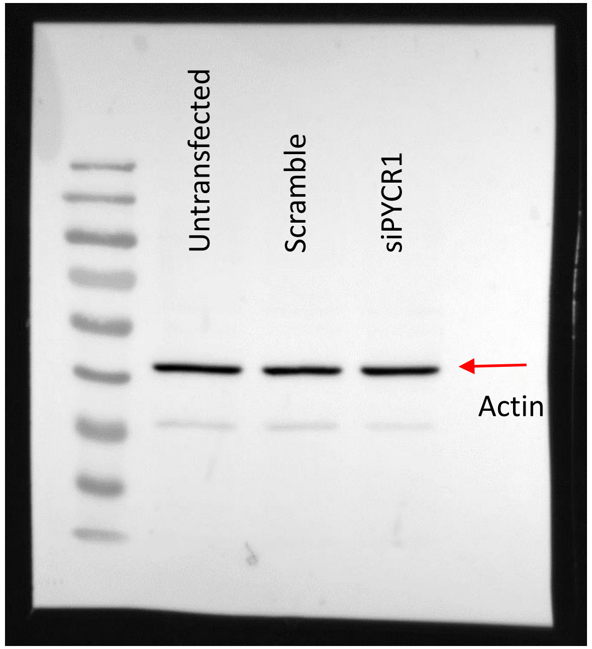

Original western blot images used in figures

FIGURE 4E

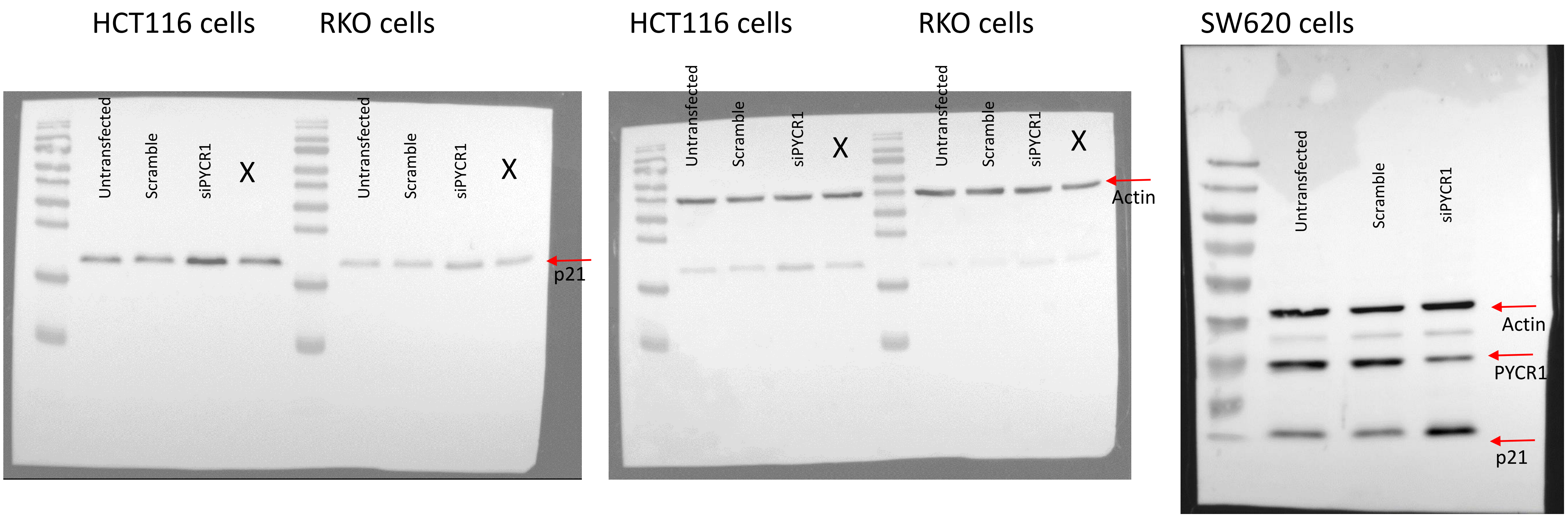

Original western blot images used in figures

FIGURE 5C

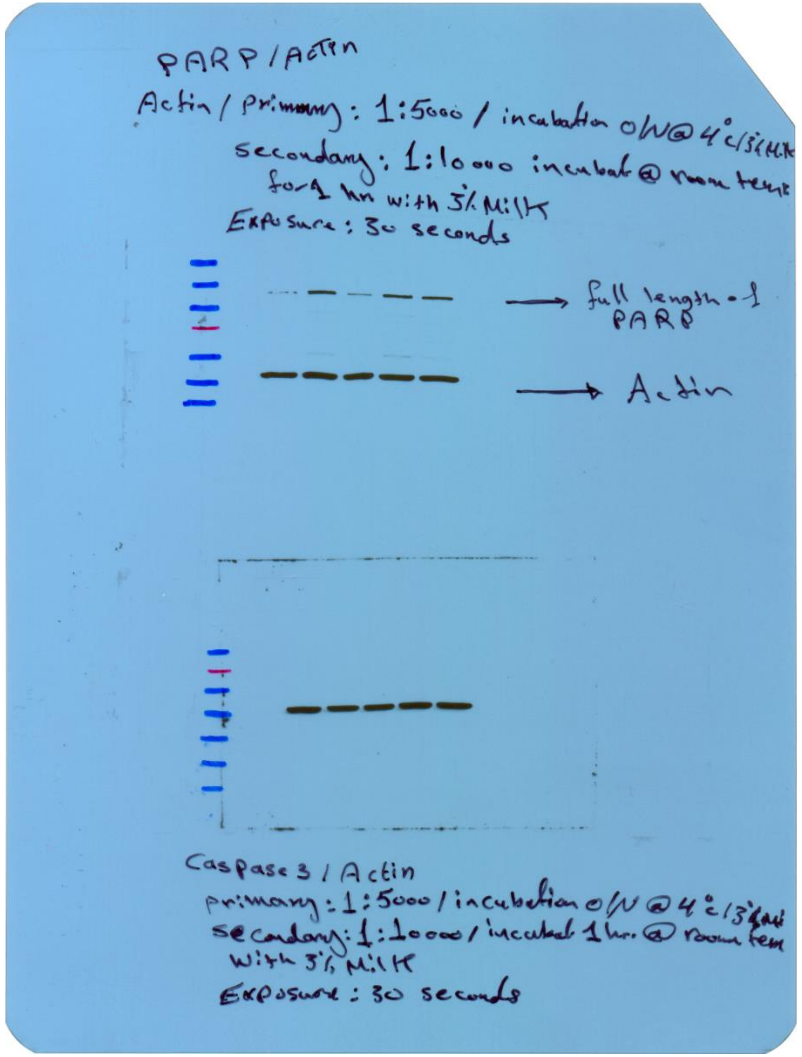

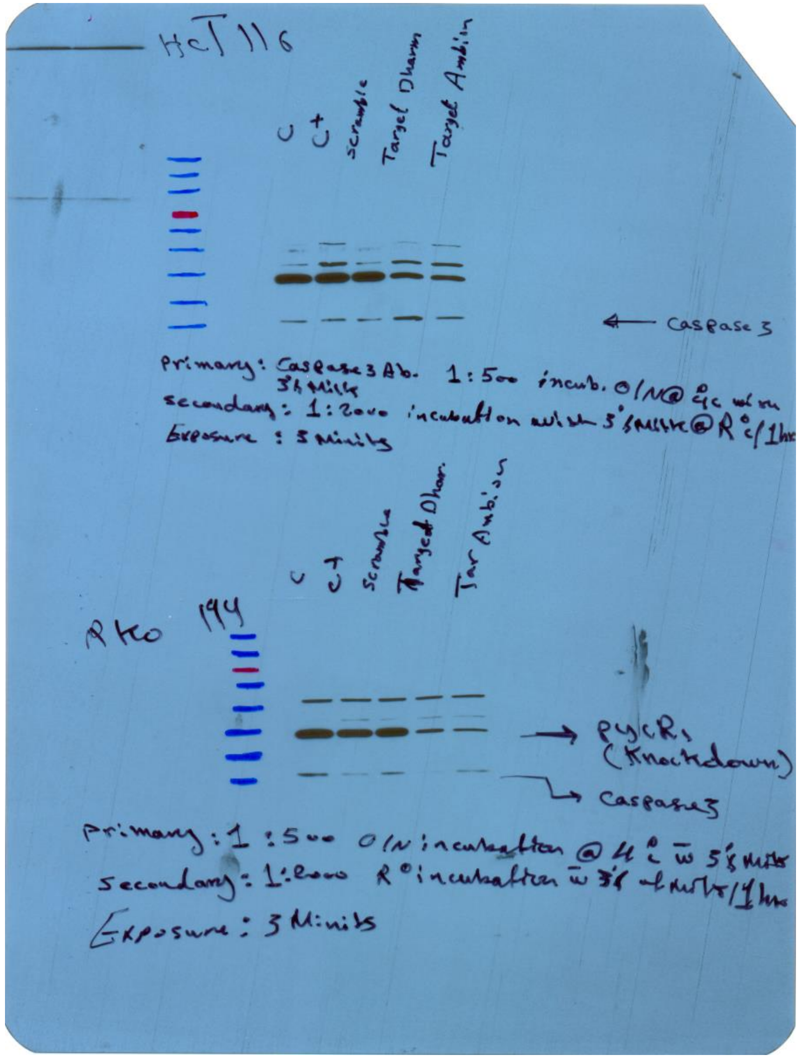

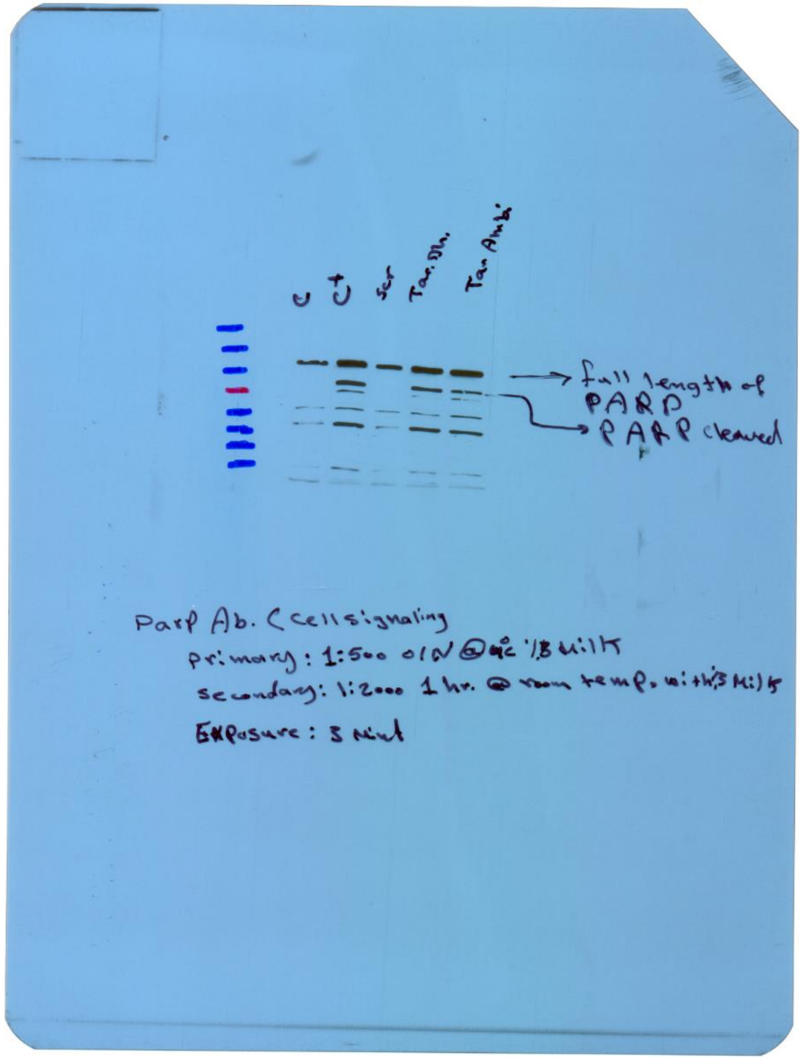

HCT116 cells

Original western blot images used in figures

FIGURE 5D

BAK

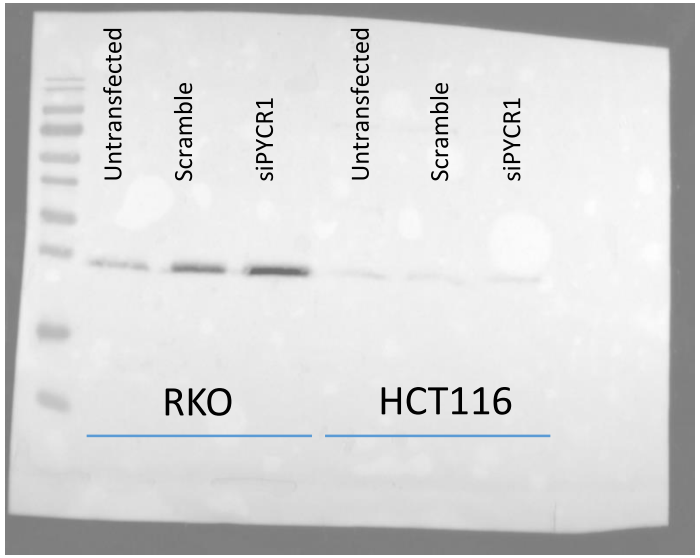

PUMA

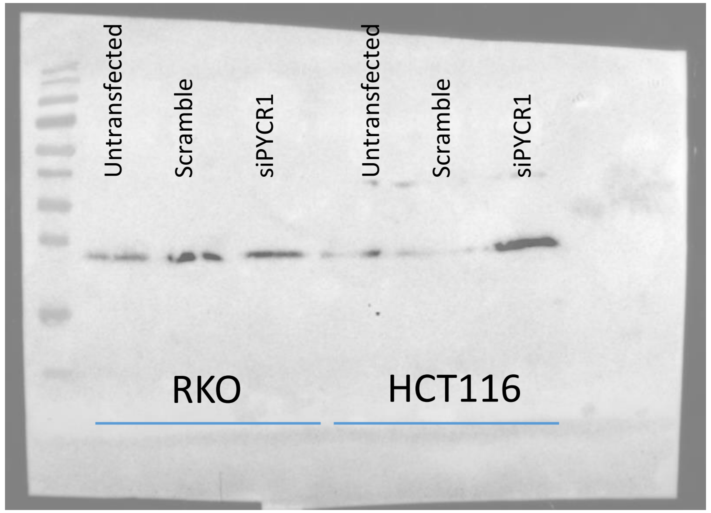

BAK membrane – tubulin antibody

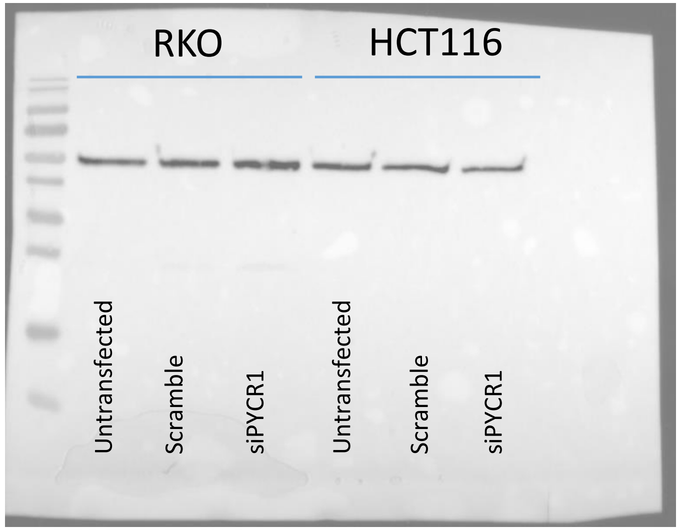

PUMA membrane – tubulin antibody

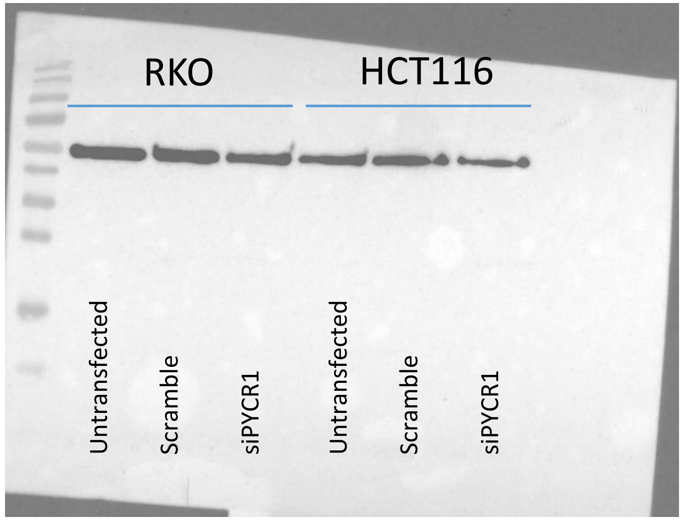

Original western blot images used in figures

FIGURE 5D

BCL2

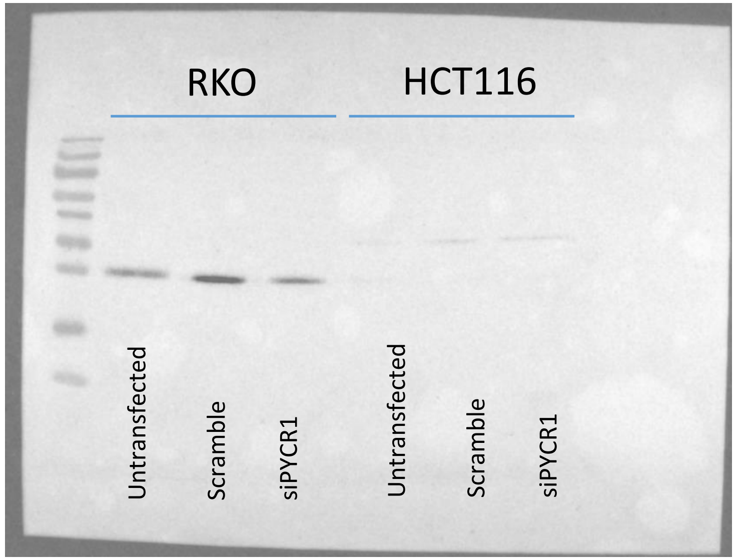

BAX

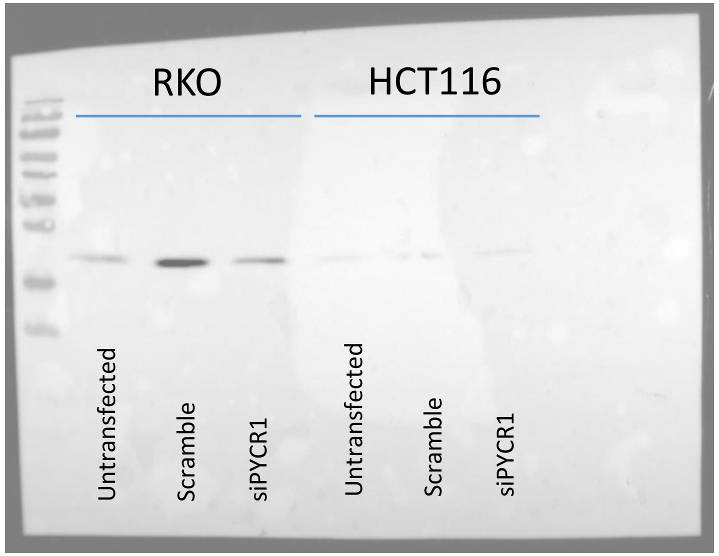

BCL2 membrane – tubulin antibody

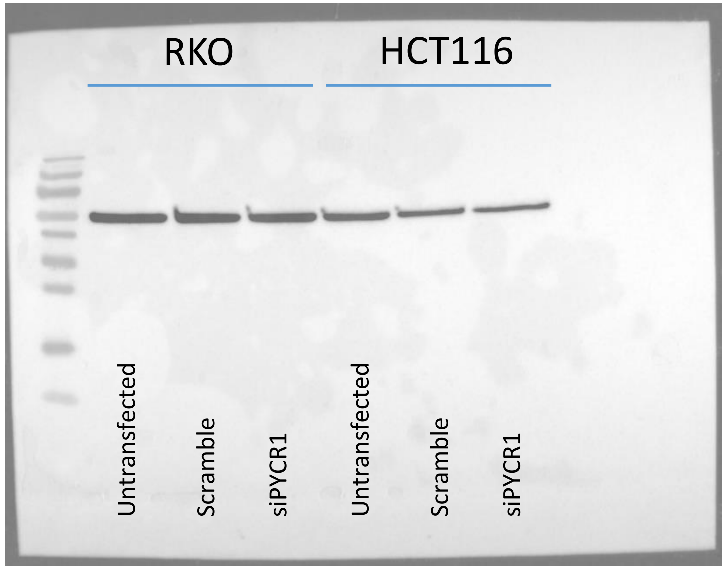

BAX membrane – tubulin antibody

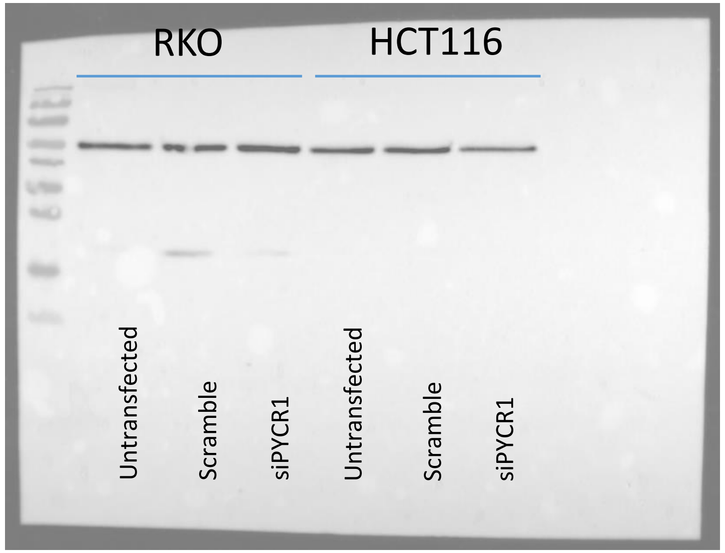

Original western blot images used in figures

FIGURE 6E

RKO cells

Cyclin D3/Actin

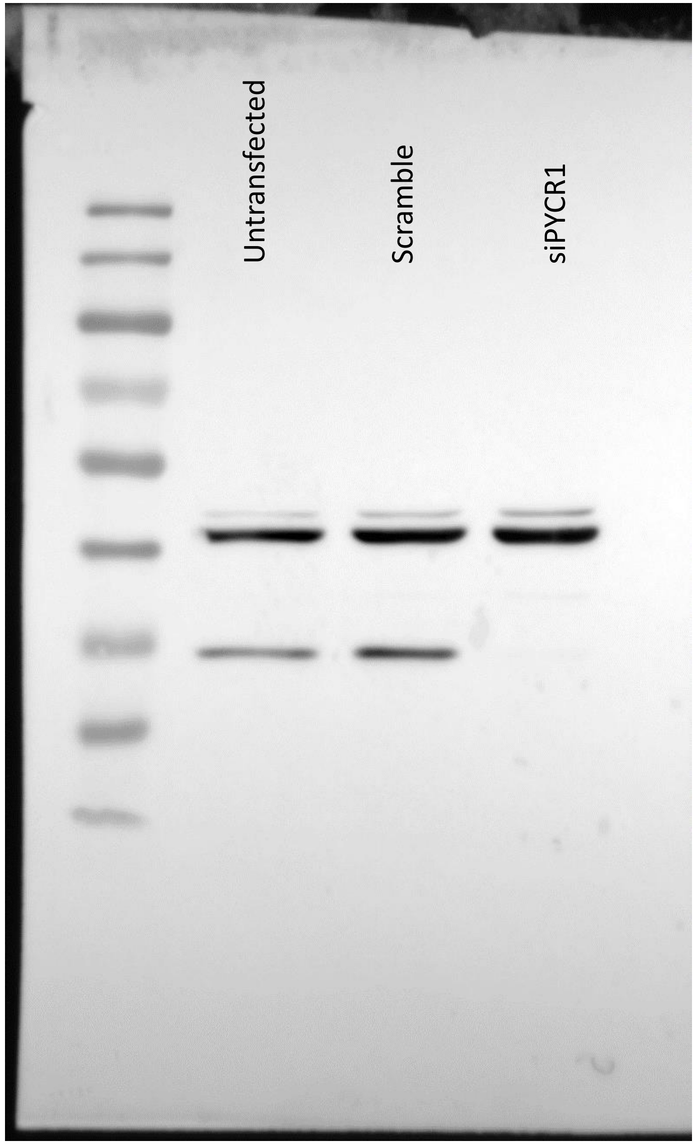

Cyclin D1

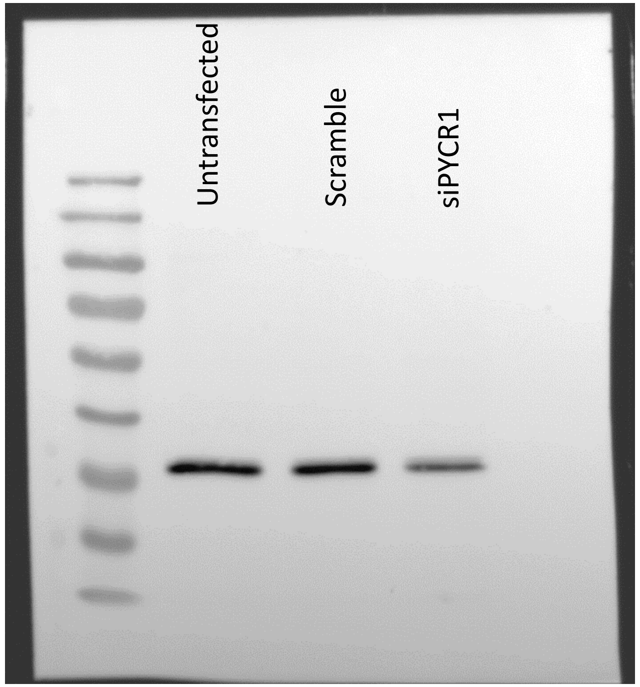

Original western blot images used in figures

FIGURE 6F

HCT 116 cells

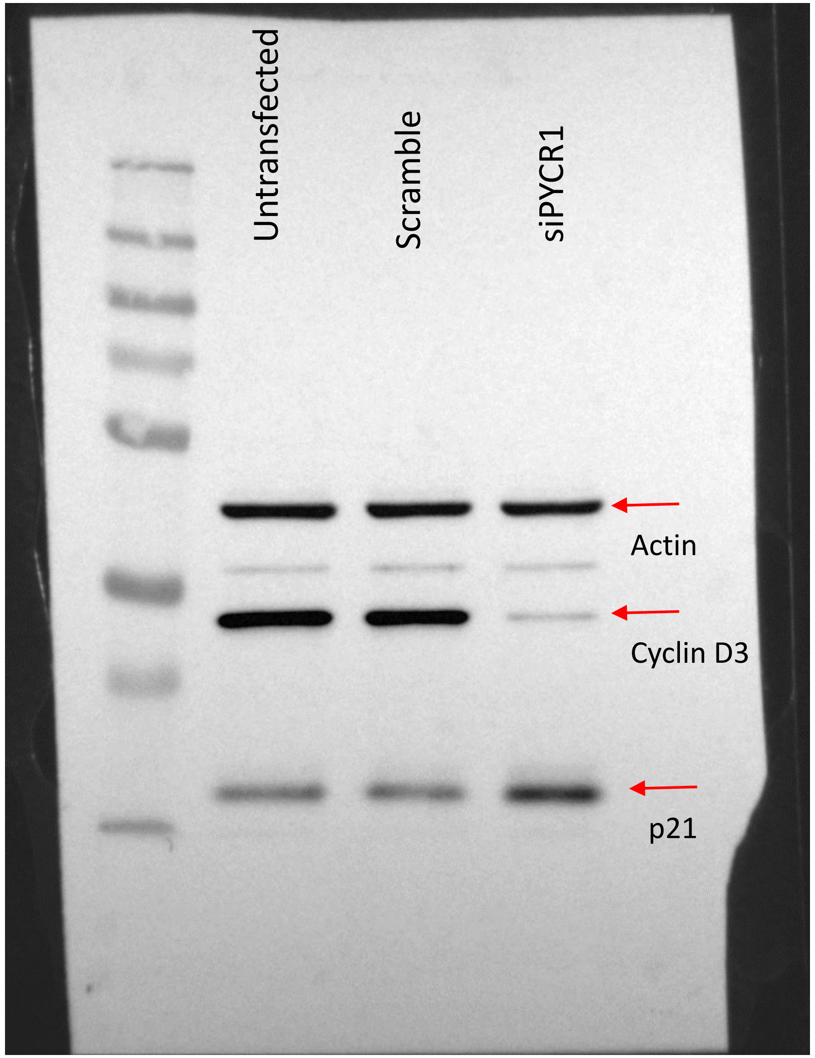

Original western blot images used in figures

FIGURE 6G

Untransfected Scramble siPYCR1

Cyclin D1 Cyclin D3 Actin

SW620 cells

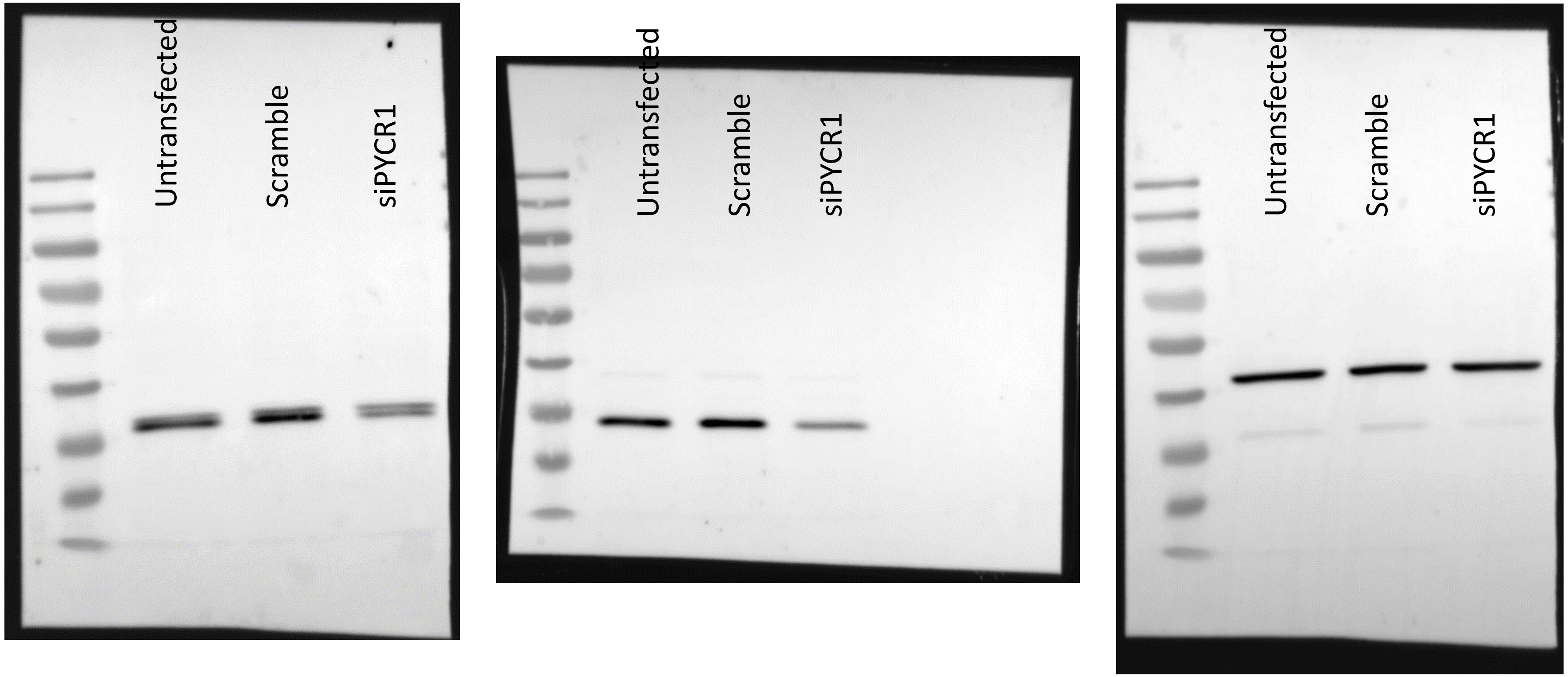

Original western blot images used in figures

FIGURE 7A

PYCR1

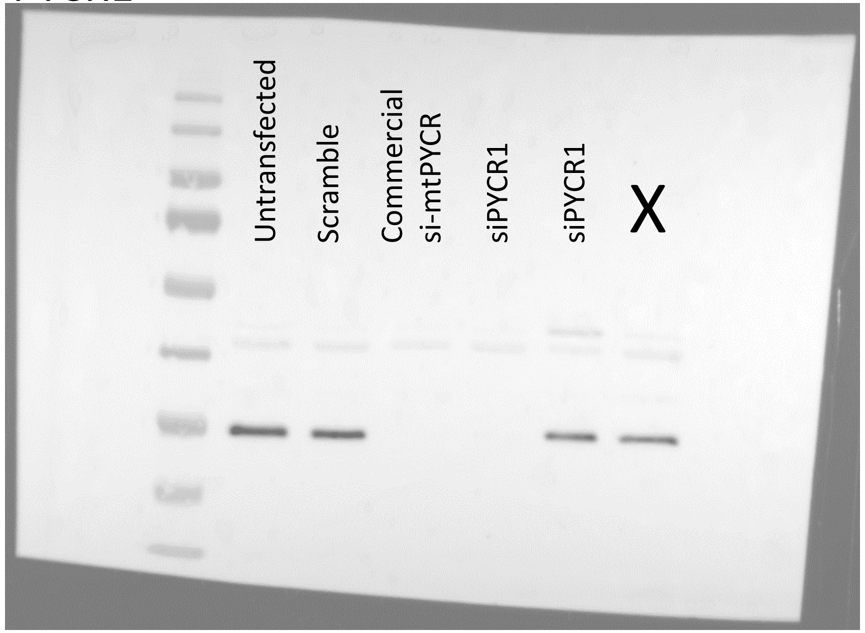

PYCR2

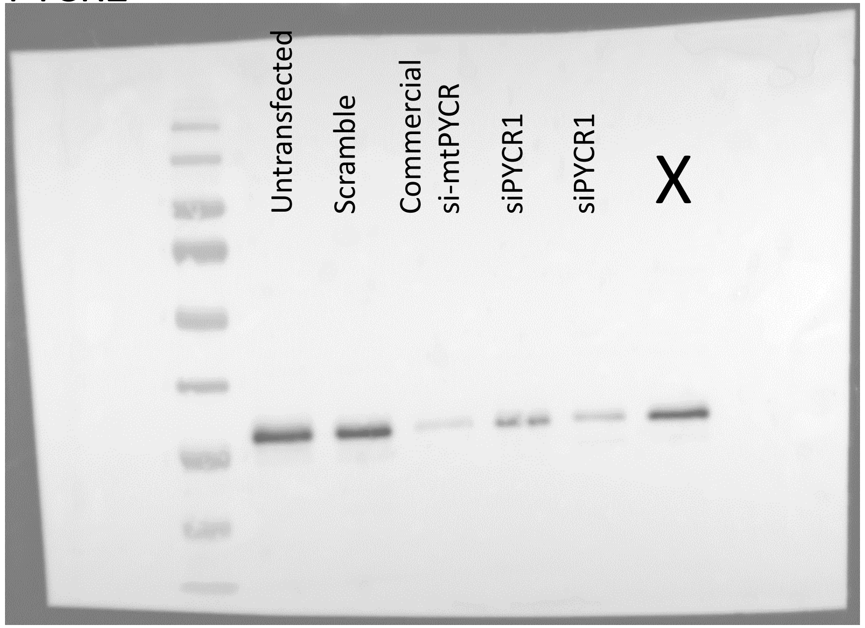

HCT116 Cells

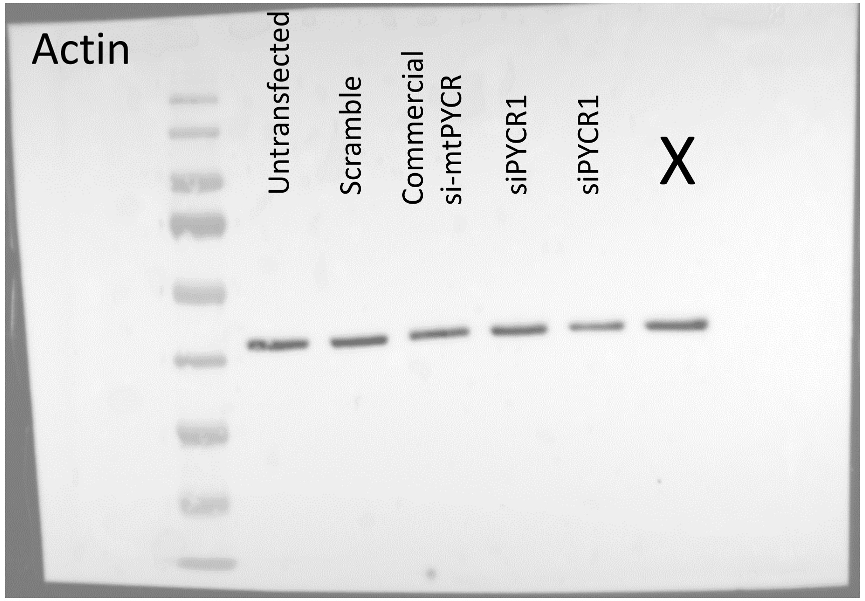

Original western blot images used in figures

FIGURE 7C

PYCR1

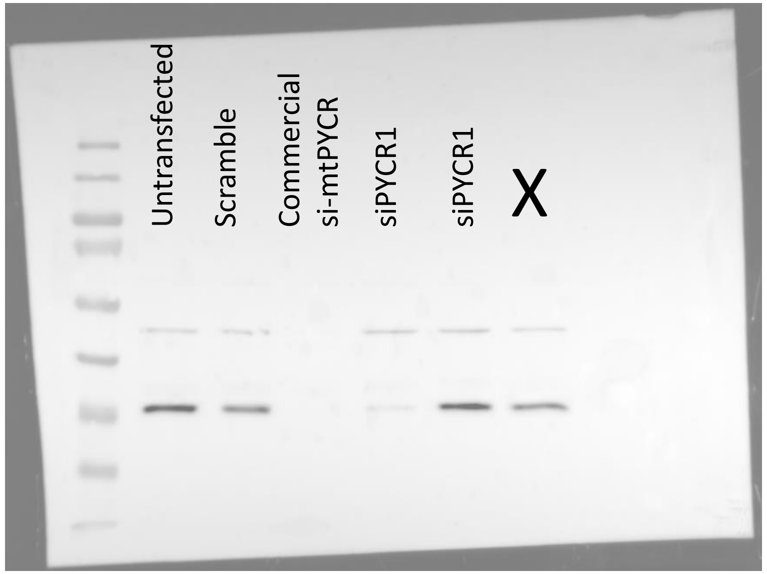

PYCR2

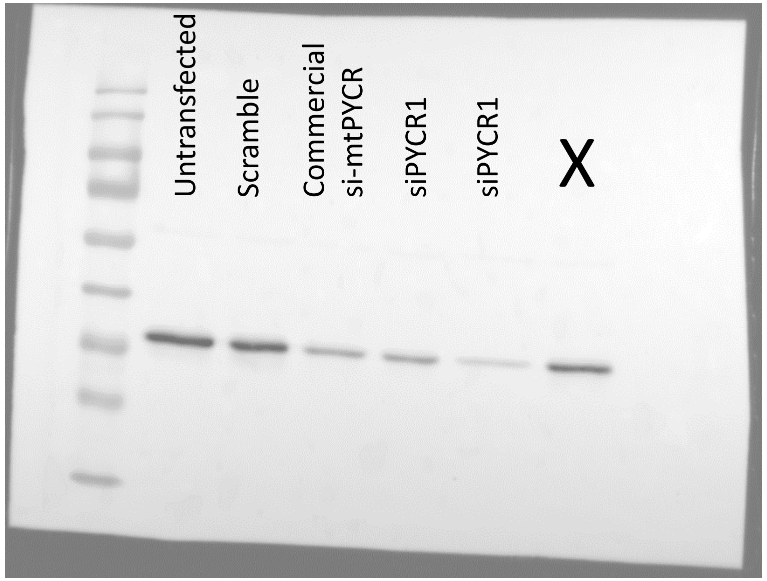

RKO Cells

Actin

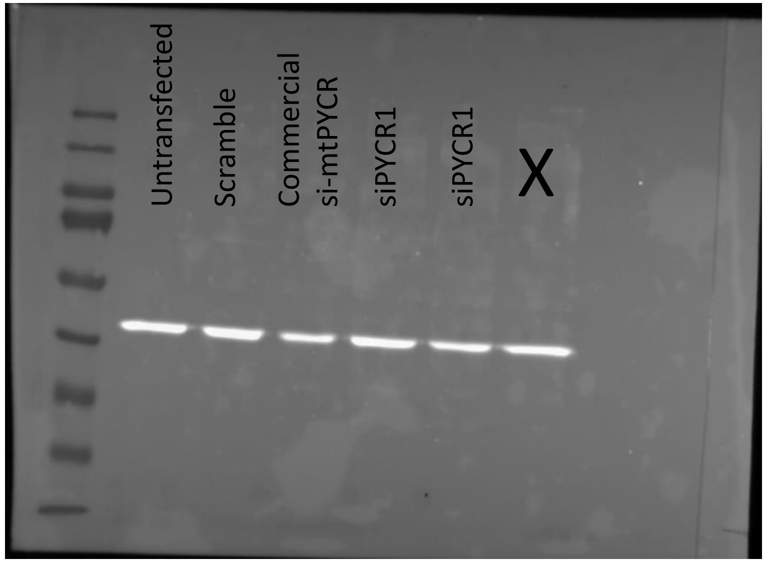

Actin

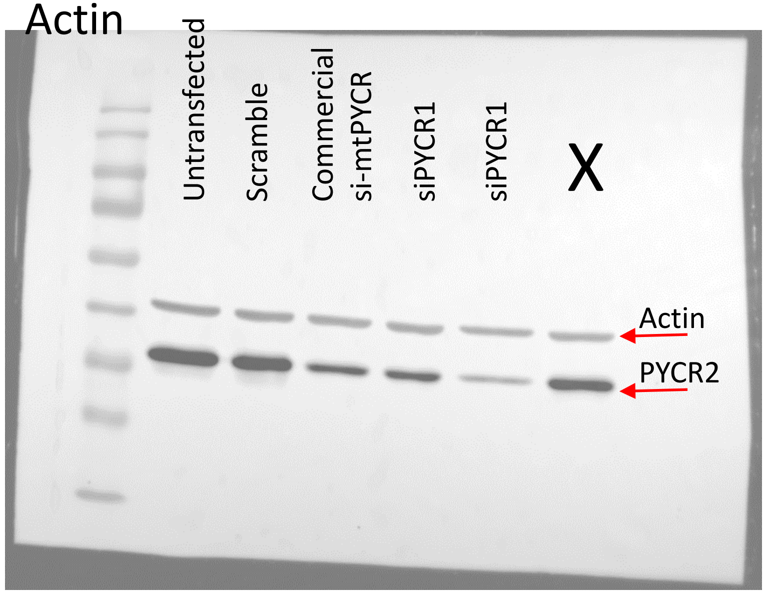

Original western blot images used in figures

FIGURE SUPPL 5

Panel C

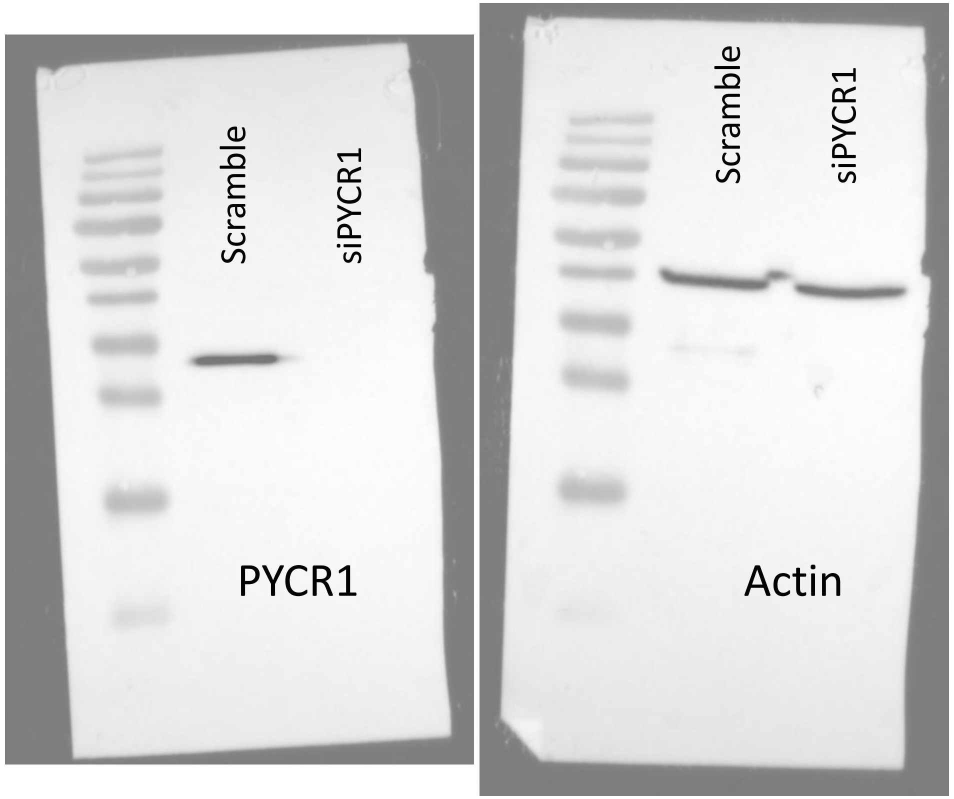

RKO cells in HPLM

Panel D

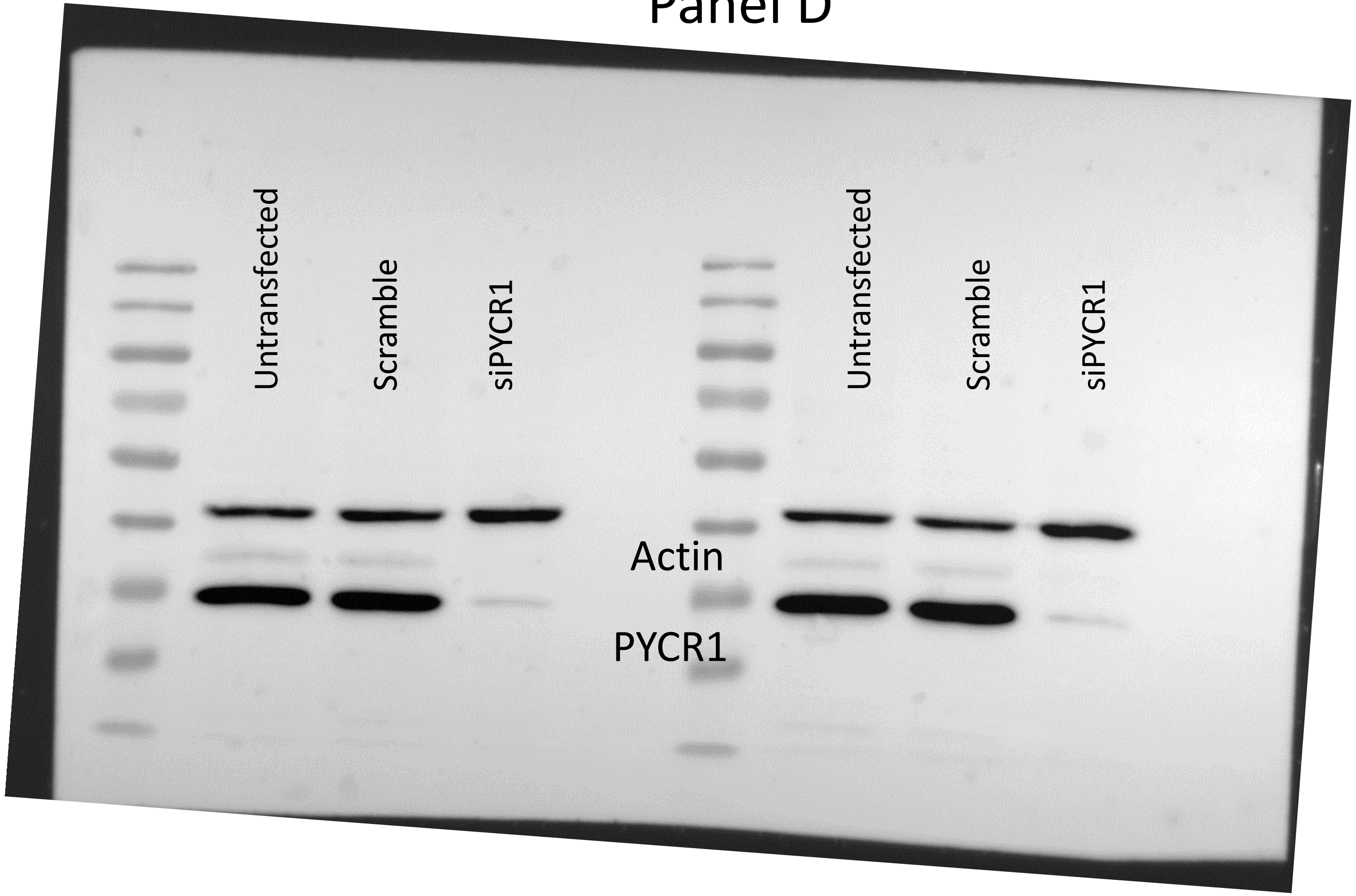

HCEC cells

Original western blot images used in figures

FIGURE SUPPL 6A

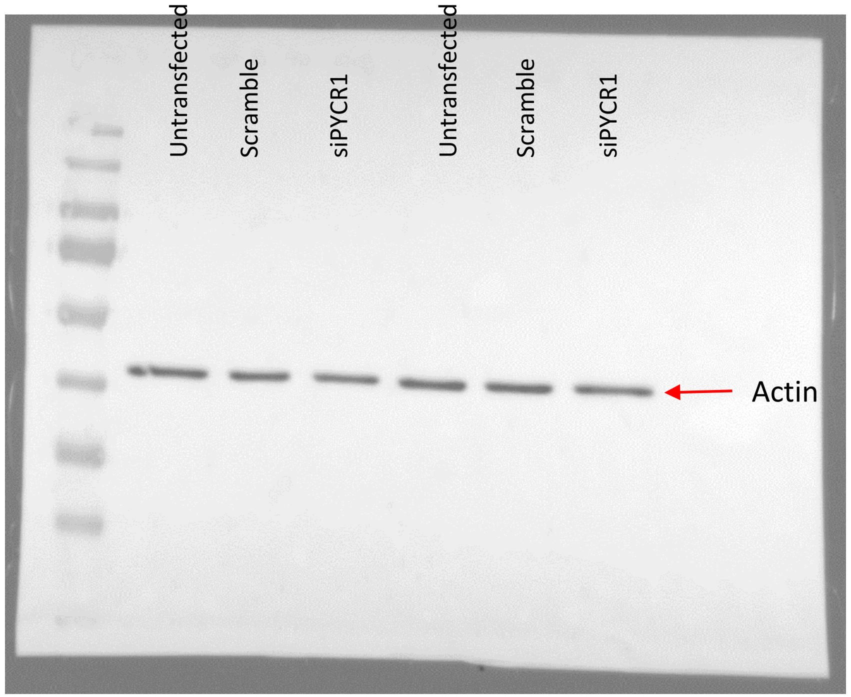

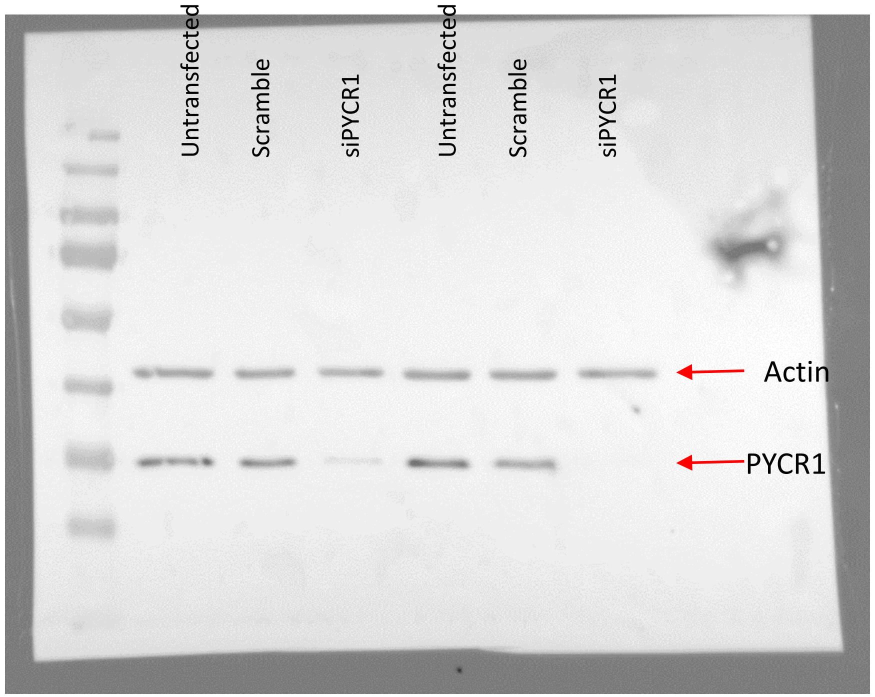

Original western blot images used in figures

FIGURE SUPPL 6C

Tubulin Total-4EBP1

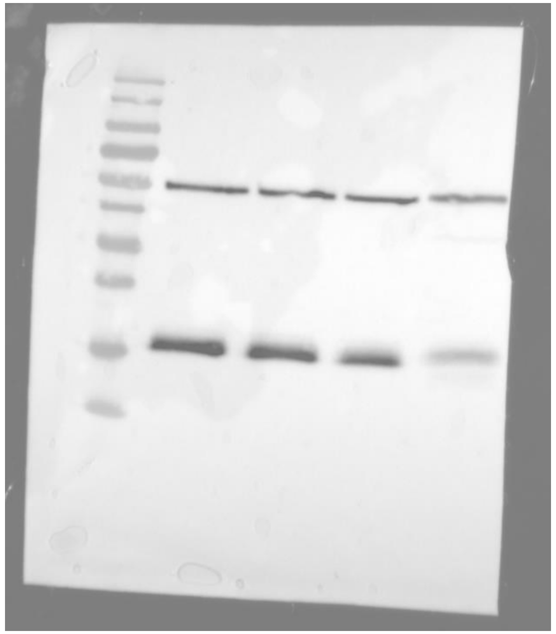

Total-4EBP1

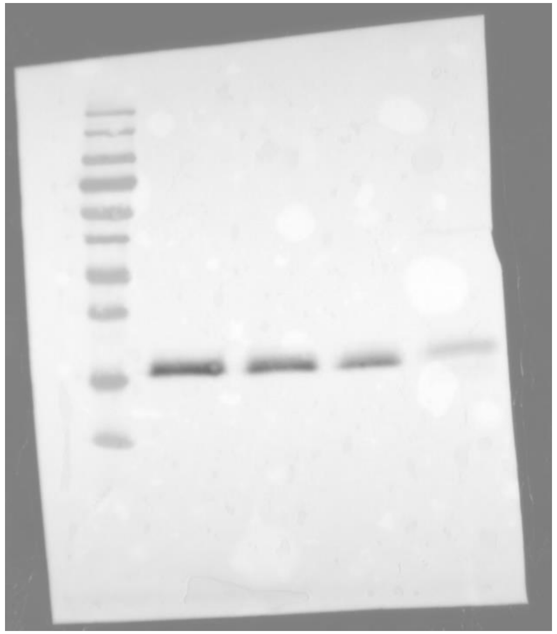

phospho-4EBP1

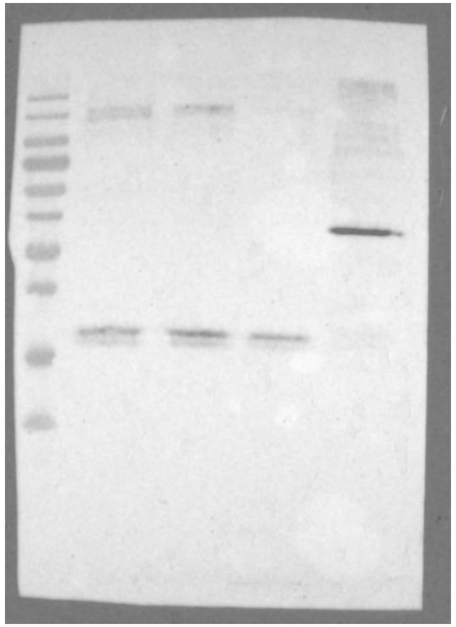

Tubulin phospho-4EBP1

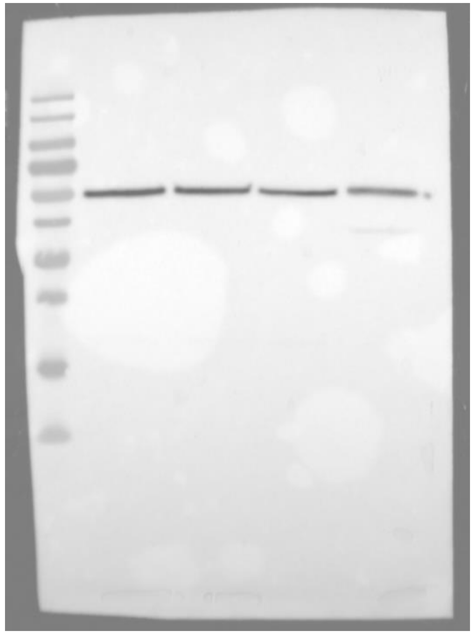

Original western blot images used in figures

FIGURE SUPPL 6C

Phospho-eif2a

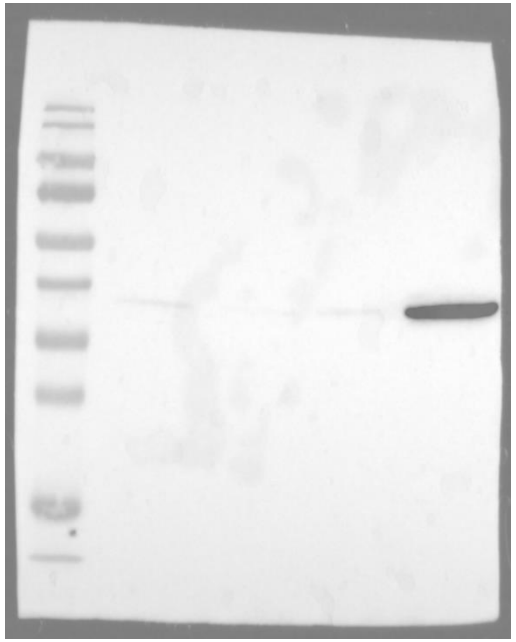

Tubulin

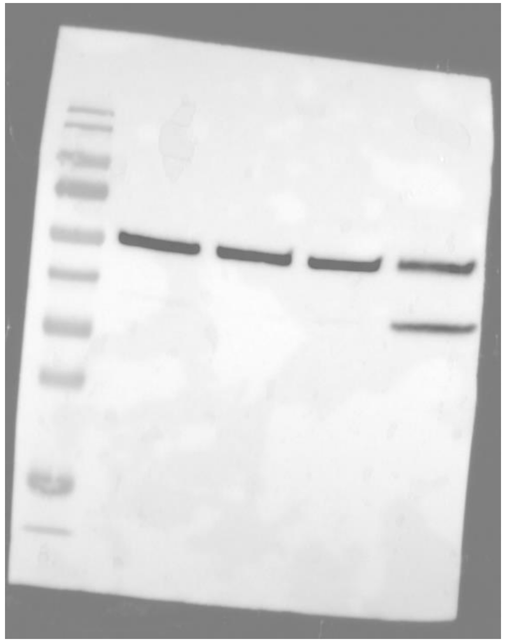

Total-eif2a

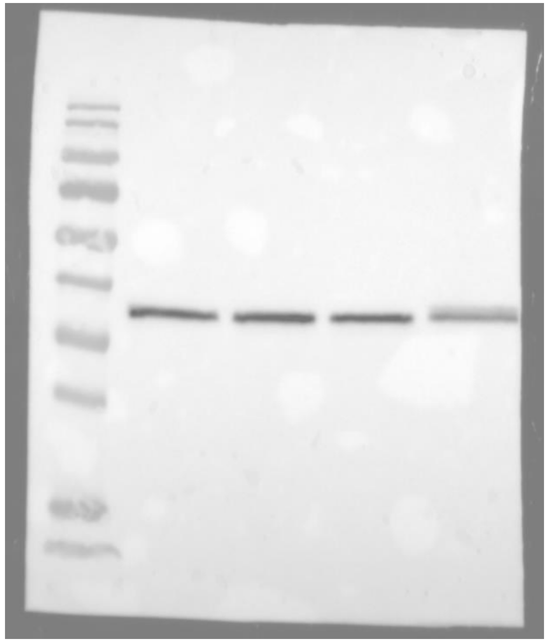

Tubulin

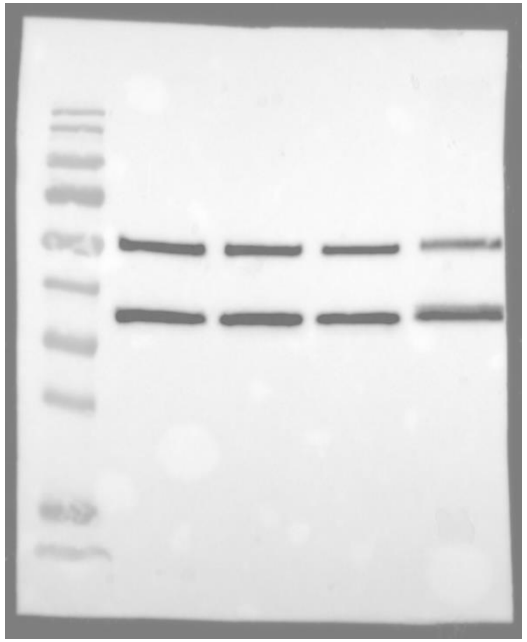

Original western blot images used in figures

FIGURE SUPPL 6C

Tubulin Total-p70S6K

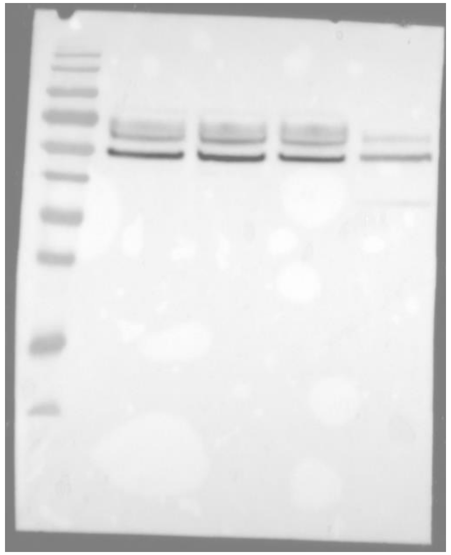

Total-p70S6K

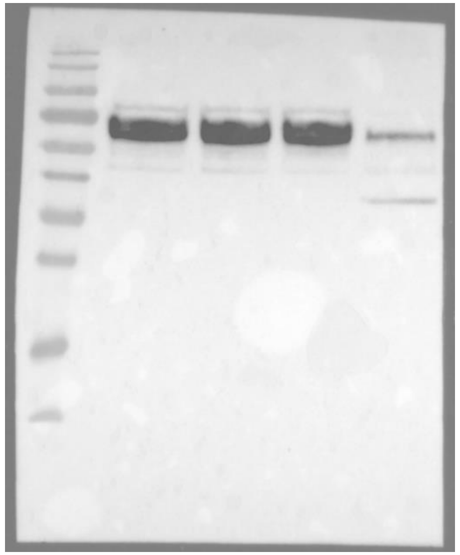

phospho-p70S6K

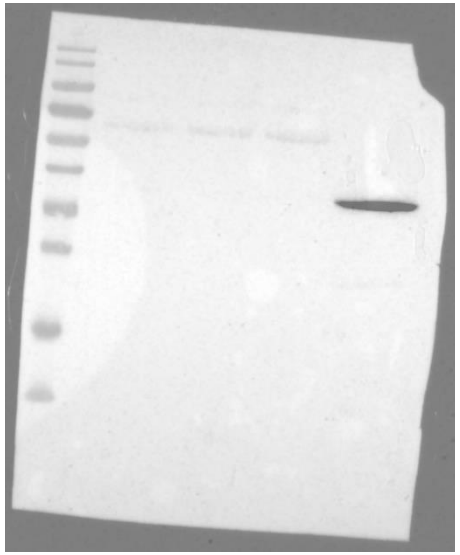

Tubulin phospho-p70S6K

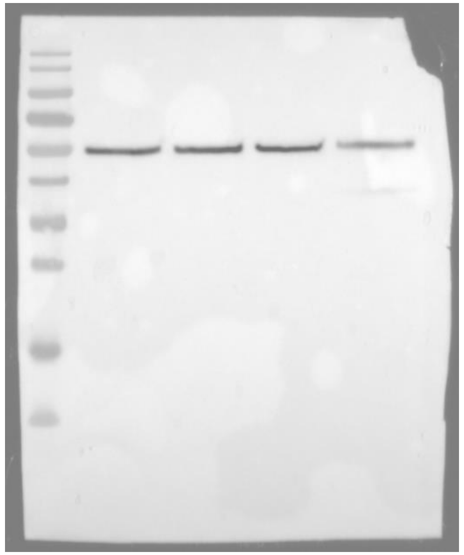

Original western blot images used in figures

FIGURE SUPPL 6F

p-p38

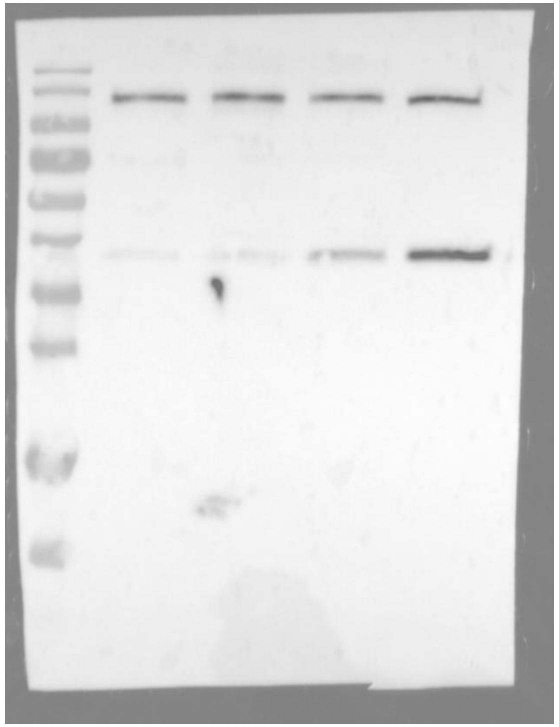

p-p38 blot- tubulin

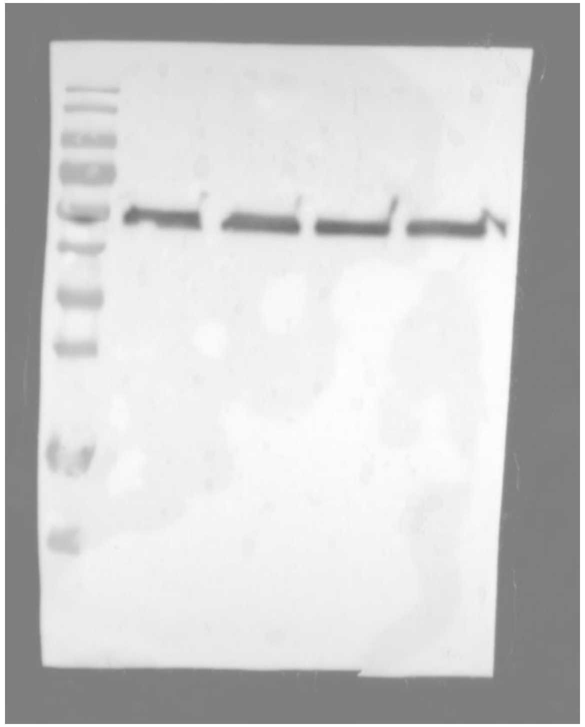

p38

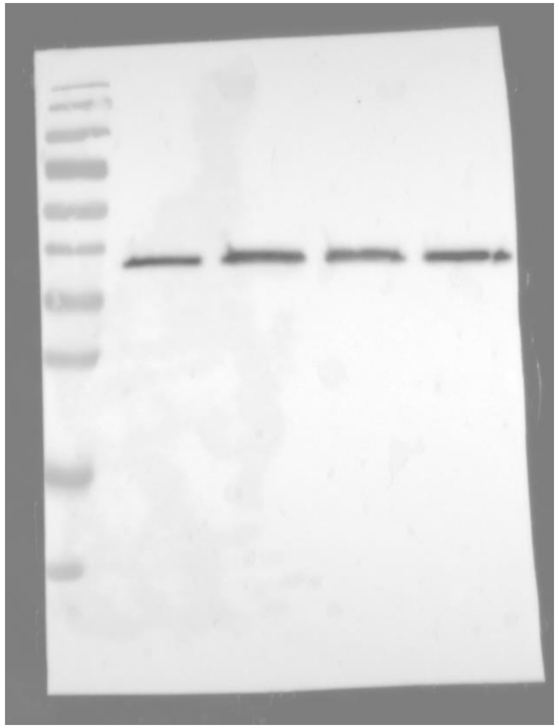

P38 blot -tubulin

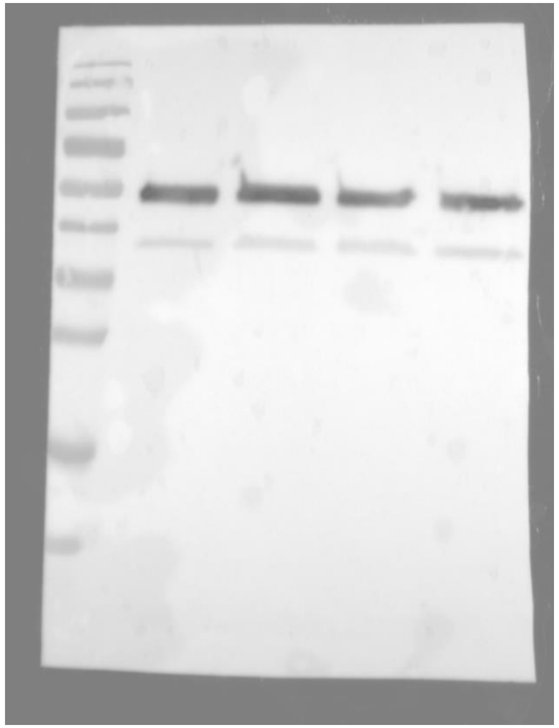

RKO

Original western blot images used in figures

FIGURE SUPPL 6F

phospho-p38

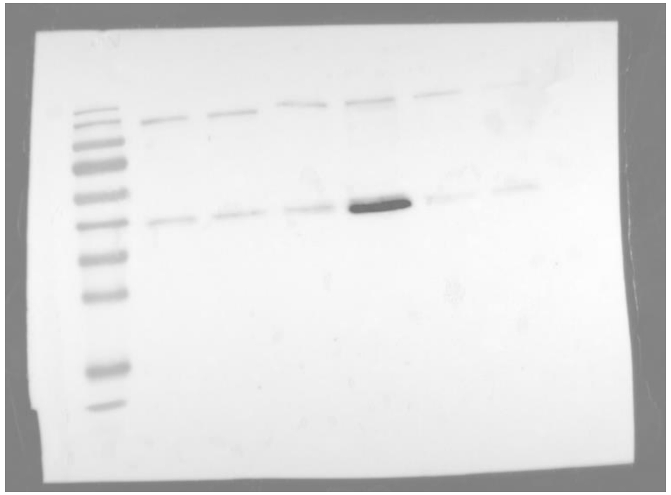

phospho-p38 blot- tubulin

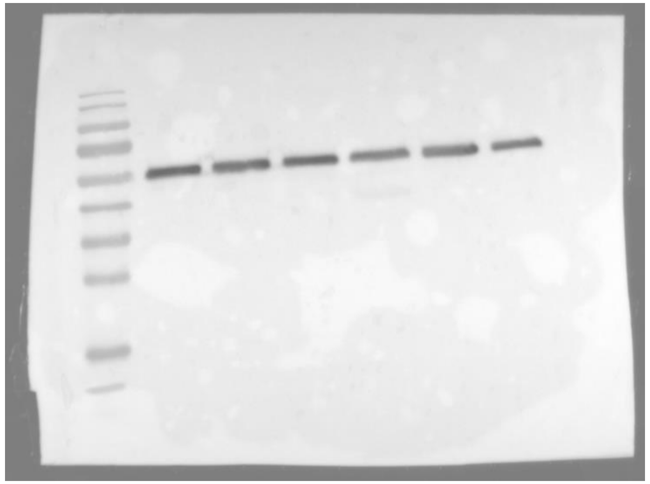

p38

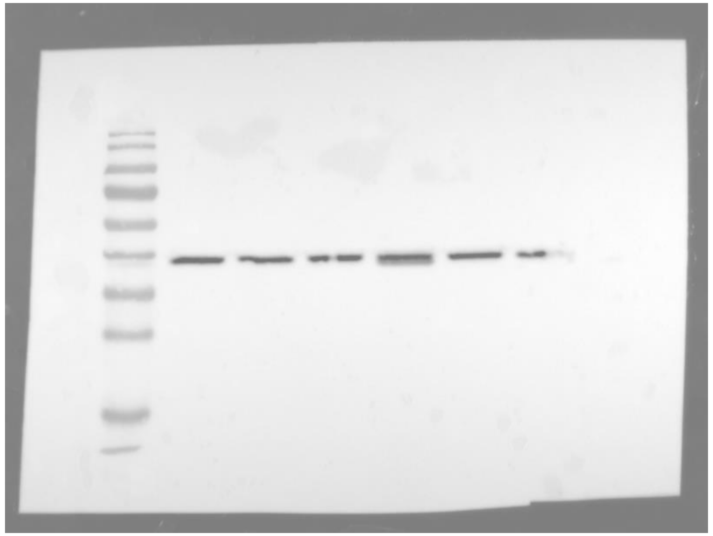

P38 blot -tubulin

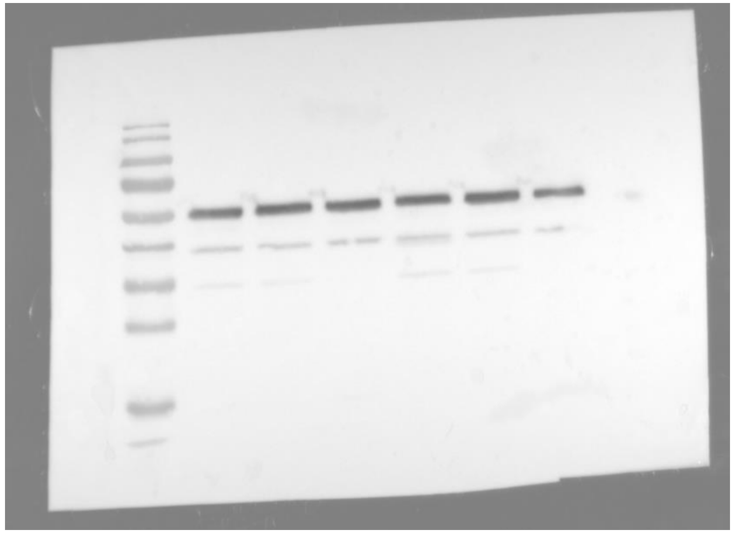

HCT116
